# Supplementary material for: From network to phenotype: the dynamic wiring of an Arabidopsis transcriptional network induced by osmotic stress
Source: Mol Syst Biol. 2017 Dec 21;13(12):961. doi: 10.15252/msb.20177840 (PMC5740496; doi:10.15252/msb.20177840)
Supplement: Supplementary file 1 — Appendix [file MSB-13-961-s001.pdf]

Content Appendix

Appendix Figure S1	The induction of candidate transcription factors, potentially involved in leaf growth regulation upon mild osmotic stress
Appendix Table S1	List of 20 genes encoding transcription factors of the putative mannitol-responsive GRN
Appendix Figure S2	Expression profiles 12 h, 16 h, 24 h and 48 h after transfer to mannitol to evaluate the steady state of every transcription factor
Appendix Figure S3	Overview of experimental data related to ERF-1 (AT4G17500)
Appendix Figure S4	Overview of experimental data related to ERF2 (AT5G47220)
Appendix Figure S5	Overview of experimental data related to ERF5 (AT5G47230)
Appendix Figure S6	Overview of experimental data related to ERF6 (AT4G17490)
Appendix Figure S7	Overview of experimental data related to ERF8 (AT1G53170)
Appendix Figure S8	Overview of experimental data related to ERF9 (AT5G44210)
Appendix Figure S9	Overview of experimental data related to ERF11 (AT1G28370)
Appendix Figure S10	Overview of experimental data related to ERF59 (AT1G06160)
Appendix Figure S11	Overview of experimental data related to ERF98 (AT3G23230)
Appendix Figure S12	Overview of experimental data related to RAP2.6L (AT5G13330)
Appendix Figure S13	Overview of experimental data related to STZ (AT1G27730)
Appendix Figure S14	Overview of experimental data related to ZAT6 (AT5G04340)
Appendix Figure S15	Overview of experimental data related to MYB51 (AT1G18570)
Appendix Figure S16	Overview of experimental data related to WRKY6 (AT1G62300)
Appendix Figure S17	Overview of experimental data related to WRKY15 (AT2G23320)
Appendix Figure S18	Overview of experimental data related to WRKY28 (AT4G18170)
Appendix Figure S19	Overview of experimental data related to WRKY30 (AT5G24110)
Appendix Figure S20	Overview of experimental data related to WRKY33 (AT2G38470)
Appendix Figure S21	Overview of experimental data related to WRKY40 (AT1G80840)
Appendix Figure S22	Overview of experimental data related to WRKY48 (AT5G49520)
Appendix Figure S23	The differential expression of 20 genes encoding transcription factors upon cycloheximide treatment
Appendix Table S2	Overview of the GOF lines used for the large-scale expression analysis and the crosses
Appendix Figure S24	Transient expression assays to assess the effect of adding a GR-domain
Appendix Table S3	The confirmed regulatory interactions between 20 genes encoding transcription factors
Appendix Table S4	The additional confirmed regulatory interactions between 20 genes encoding transcription factors as a result of the combination of two transcription factors
Appendix Figure S25	Five different effects of two TFs on a target gene and the estimated position of the DNA-binding elements on the target promoter

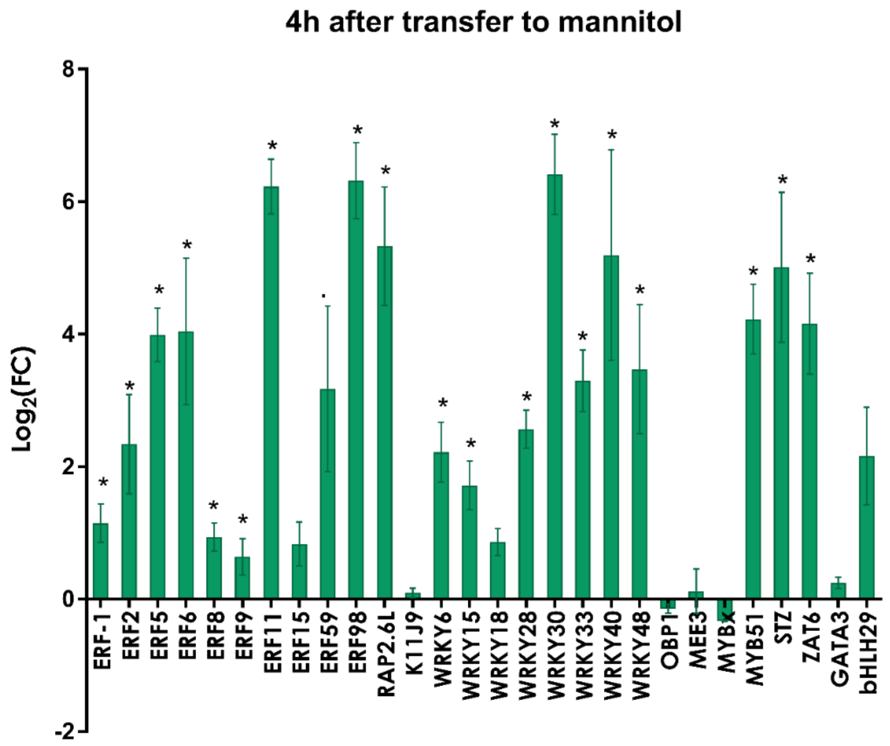

Appendix Figure S1 - The induction of candidate transcription factors, potentially involved in leaf growth regulation upon mild osmotic stress.

Wild-type plants were grown on ½MS medium covered with a nylon mesh and after 15 days, the plants were transferred to control or 25 mM mannitol-containing medium. After 4 h, the third leaf was harvested and the expression levels of 28 transcription factors was measured.

Data information: data are presented as mean ± SEM. FC = Fold change, n = 3 independent experiments. * = P < 0.05, · = P < 0.1, unpaired two-sided Student's t-test.

Appendix Table S1 – List of 20 genes encoding transcription factors of the putative mannitol-responsive GRN.

The table includes the AT-code, family, subfamily, domains (CM=Conserved Motif, WD=WRKY Domain), the reference in which the mutant line was previously described, literature references, the earliest time point when the gene was significantly upregulated (FDR<0.05) upon osmotic stress (Skirycz et al., 2011) and the log_2(fold change) values after 4 h of induced *ERF6* overexpression (Dubois et al., 2013).

AT-Code	Gene ID	Family	Sub-family	Domains	Skyricz et al	Dubois et al	Described mutant	References
**AT4G17500**	***ERF-1***	AP2/ERF	IXa	CMIX-2, CMIX-3				Etchells et al., 2012 Shin et al., 2010
**AT5G47220**	***ERF2***	AP2/ERF	IXa	CMIX-2, CMIX-3	24 h		Dubois et al., 2016	Kouno et al., 2012 McGrath et al., 2005
**AT5G47230**	***ERF5***	AP2/ERF	IXb	CMIX-2, CMIX-5	12 h		Dubois et al., 2013	Son et al., 2012 Moffat et al., 2012 Dubois et al., 2013
**AT4G17490**	***ERF6***	AP2/ERF	IXb	CMIX-2, CMIX-5			Dubois et al., 2013	Son et al., 2012 Moffat et al., 2012 Dubois et al., 2013
**AT1G53170**	***ERF8***	AP2/ERF	VIIIa	CMVIII-1 (EAR), CMVIII-2	24 h		Dubois et al., 2016	Koyama et al., 2013
**AT5G44210**	***ERF9***	AP2/ERF	VIIIa	CMVIII-1 (EAR)	24 h		Maruyama et al., 2013	Ogata et al., 2015 Maruyama et al., 2013 Camehl er al., 2010
**AT1G28370**	***ERF11***	AP2/ERF	VIIIa	CMVIII-1 (EAR), CMVIII-2	24 h		Dubois et al., 2015	Tsai et al., 2014 Dubois et al., 2013 Li et al., 2013
**AT1G06160**	***ERF59/ ORA59***	AP2/ERF	IXc	CMIX-1, CMIX-4	12 h	2.950	Zander et al., 2014	Van der Does et al., 2013 Zarei et al., 2011 Pré et al., 2008
**AT3G23230**	***ERF98***	AP2/ERF	IXc	CMIX-1	24 h	0.767	Zhang et al., 2012	Tiwari et al., 2012 Zhang, Wang et al., 2012 Ou et al., 2011 Çevik, Kidd et al., 2012
**AT5G13330**	***RAP2.6L***	AP2/ERF	Xa	CMX-1	3 h		Sun et al., 2010	Efroni, Han et al., 2014 Liu, Sun et al., 2012 Asahina, Azuma et al., 2011 Krishnaswamy, Verma et al., 2011
**AT1G18570**	***MYB51***	MYB	R2R3	MYB	3 h	1.389	Gigolashvili et al., 2007	Dubois et al., 2013 Gigolashvili et al., 2007 Frerigmann et al., 2014a Frerigmann et al., 2014b
**AT1G27730**	***STZ/ZAT10***	ZFP	C1-2i	Q2-2, Q2-3, CLMLL, EAR		1.591	Mittler et al., 2006	Dubois et al., 2013 Mittler et al., 2006 Nguyen et al., 2011 Xie et al., 2012 Sakamoto et al. 2004
**AT5G04340**	***ZAT6***	ZFP	C1-2i	Q2-2, Q2-3, CLMLL, EAR	12 h	3.293	Chen et al., 2016	Liu et al., 2012 Devaiah et al., 2007 Krichevsky et al., 2006
**AT1G62300**	***WRKY6***	WRKY	IIb	WD	3 h		Kasajima et al., 2010	Chen et al., 2009 Miao et al., 2013
**AT2G23320**	***WRKY15***	WRKY	IIc	WD	24 h	1.054		Vanderauwera et al., 2012 Park et al., 2005
**AT4G18170**	***WRKY28***	WRKY	Ia	WD	12 h	1.154		Gao et al., 2013 Babitha et al., 2013
**AT5G24110**	***WRKY30***	WRKY	IIIa	WD	3 h			Scarpeci et al., 2008
**AT2G38470**	***WRKY33***	WRKY	Ib	NTWD, CTWD	12 h	0.855	Zheng et al., 2006	Dubois et al., 2013 Logemann et al., 2013 Mao et al., 2011 Jiang et al., 2009
**AT1G80840**	***WRKY40***	WRKY	IIa	WD	24 h		Xu et al., 2006	Shang et al., 2010 Jiang et al., 2010 Schweizer et al., 2013 Chen et al., 2010
**AT5G49520**	***WRKY48***	WRKY	Ib	WD	24 h		Xing et al., 2008	Xing et al., 2008

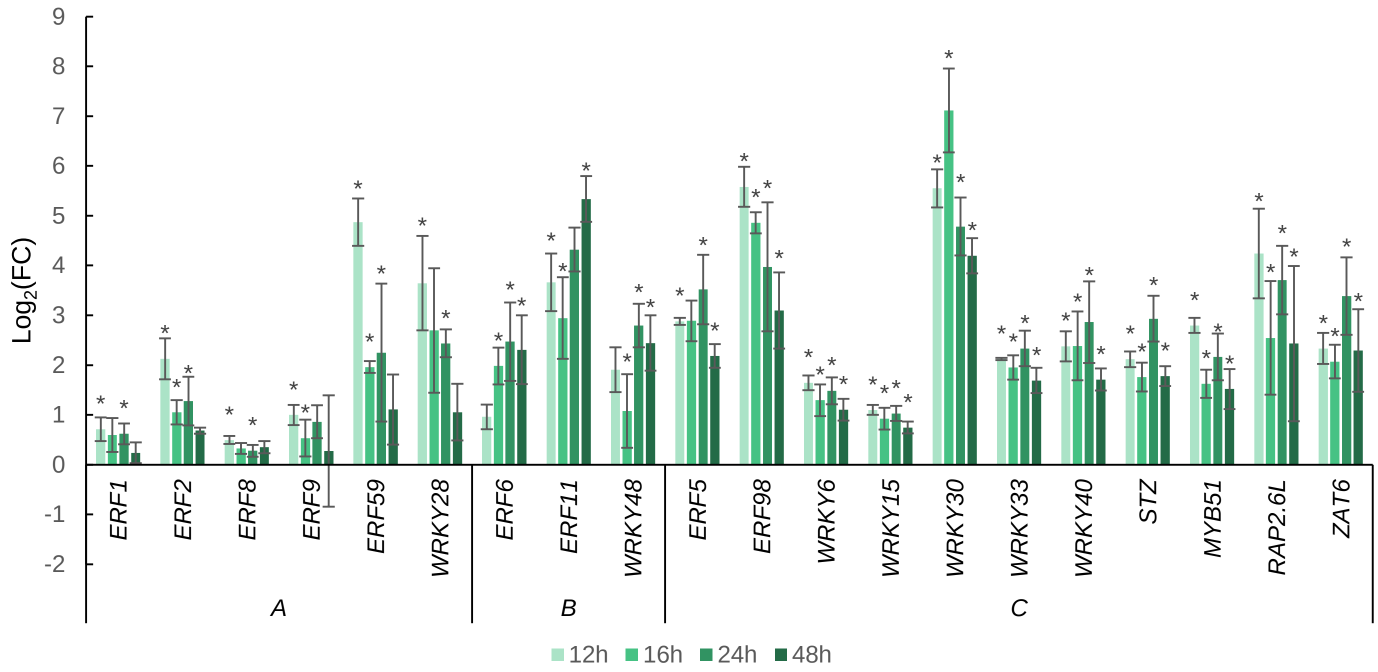

Appendix Figure S2 – Expression profiles 12 h, 16 h, 24 h and 48 h after transfer to mannitol to evaluate the steady state of every transcription factor.

The fold changes (FC) were calculated relative to the control conditions. The expression patterns were divided into three groups (A to C) based on the significance level of the last time point (48 h) and the expression profile. The expression of the transcription factor either was not significantly upregulated anymore (A), reached a minimum and increased again (B) or remained induced until 48 h after the stress (C).

Data information: data are presented as mean ± SEM. n = 4 independent experiments. * = FDR < 0.05, unpaired two-sided Student's t-test.

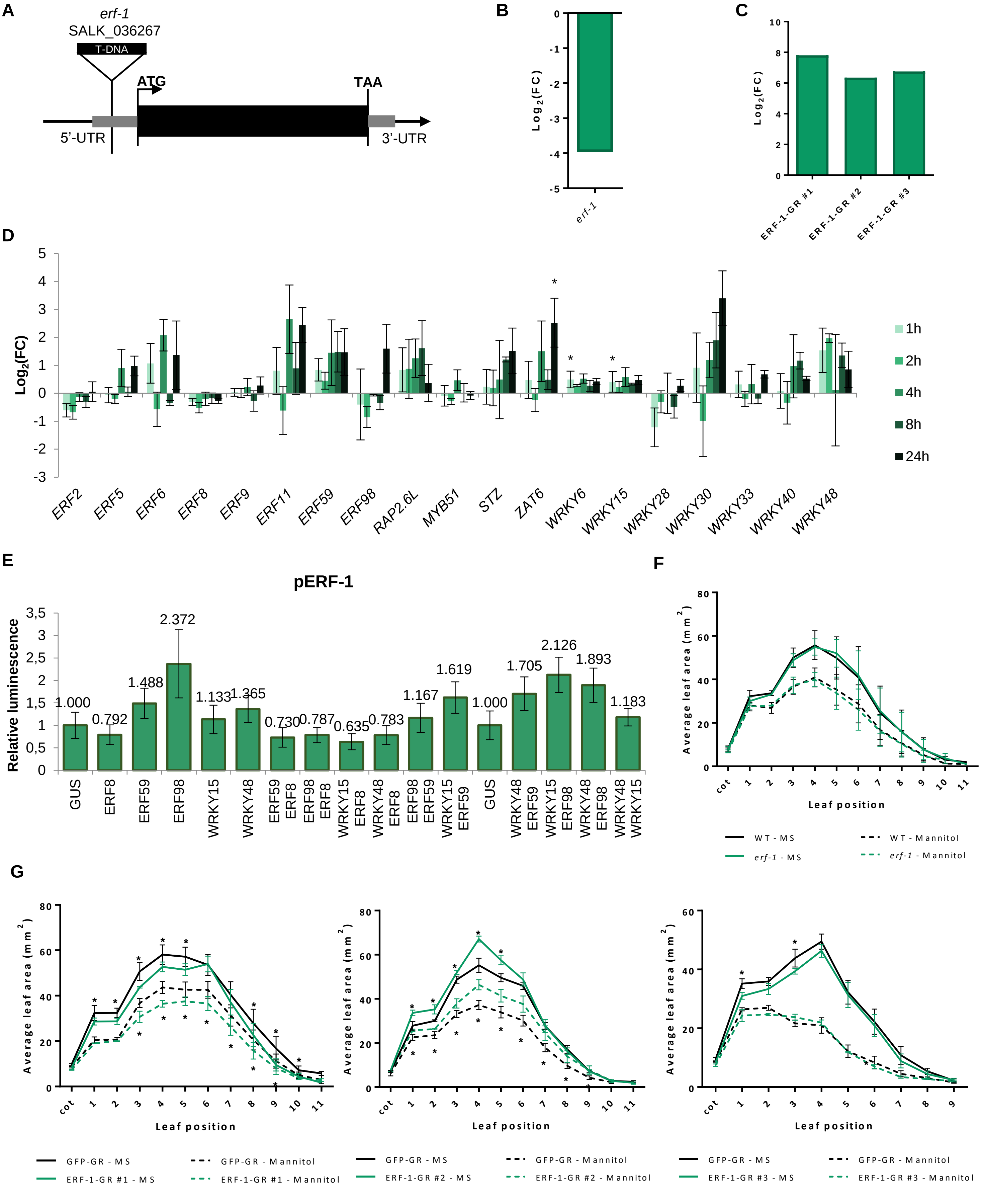

Appendix Figure S3 - Overview of experimental data related to ERF-1 (AT4G17500).

A Schematic representation of the gene with the position of the T-DNA insertion. Start and stop codon, 3'-UTR and 5'-UTR are indicated.

B,C Knock-down and overexpression measured in 10-day-old seedlings of a T-DNA insertion line (B) and three independent inducible overexpression lines (C).

D The induction of 19 genes encoding transcription factors, 1 h, 2 h, 4 h, 8 h and 24 h after transfer of the inducible overexpression line to dexamethasone-containing medium at 15 DAS.

E Activation of the ERF-1 promotor by individual or the combination of two transcription factors with transient expression assays. The presented values are luminescence levels normalized to the negative control, 35S::GUS.

F,G The area of every individual leaf was measured at 22 DAS of *erf-1* (F) and three independent lines of ERF-1-GR (G), on mannitol-containing or control MS medium (supplemented with dexamethasone in case of the inducible overexpression lines).

Data information: data are presented as mean ± SEM. FC = Fold change. n = 1 (B,C), 3 (D,E,F,G) independent experiment(s). * = FDR < 0.1 (mixed model analysis, user-defined Wald tests) (D), * = P < 0.05 (mixed model, partial F-tests) (F,G).

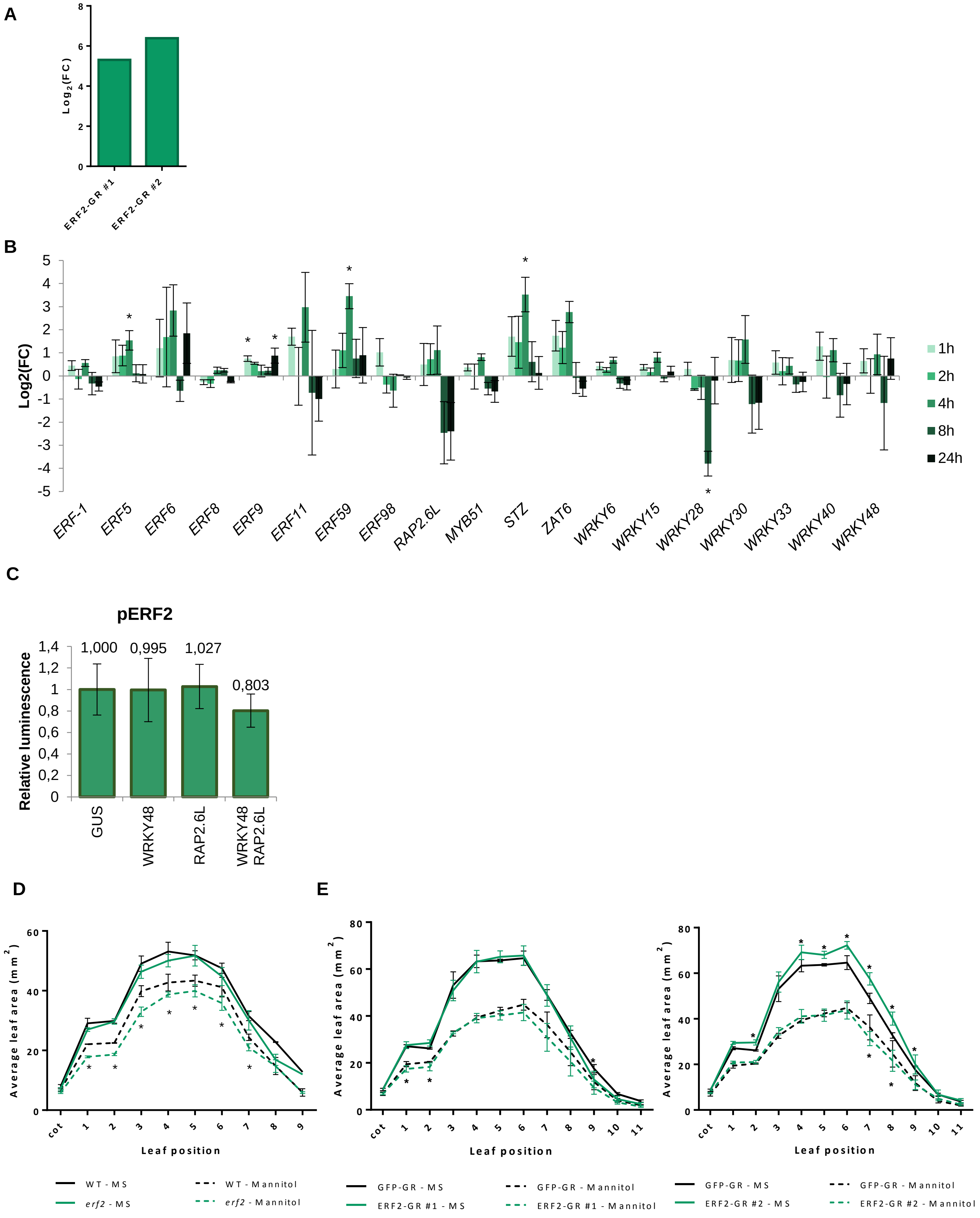

Appendix Figure S4 - Overview of experimental data related to ERF2 (AT5G47220).

A Overexpression level measured in 10-day-old seedlings of two independent inducible overexpression lines.

B The induction of 19 genes encoding transcription factors, 1 h, 2 h, 4 h, 8 h and 24 h after transfer of the inducible overexpression line to dexamethasone-containing medium at 15 DAS.

C Activation of the ERF2 promotor by individual or the combination of two transcription factors with transient expression assays. The presented values are luminescence levels normalized to the negative control, 35S::GUS.

D,E The area of every individual leaf was measured at 22 DAS of *erf2* (D) and two independent lines of ERF2-GR (E), on mannitol-containing or control MS medium (supplemented with dexamethasone in case of the inducible overexpression lines).

Data information: data are presented as mean ± SEM. FC = Fold change. n = 1 (A), 3 (B,D,E) independent experiment(s). * = FDR < 0.1 (mixed model analysis, user-defined Wald tests) (B), * = P < 0.05 (mixed model, partial F-tests) (D,E).

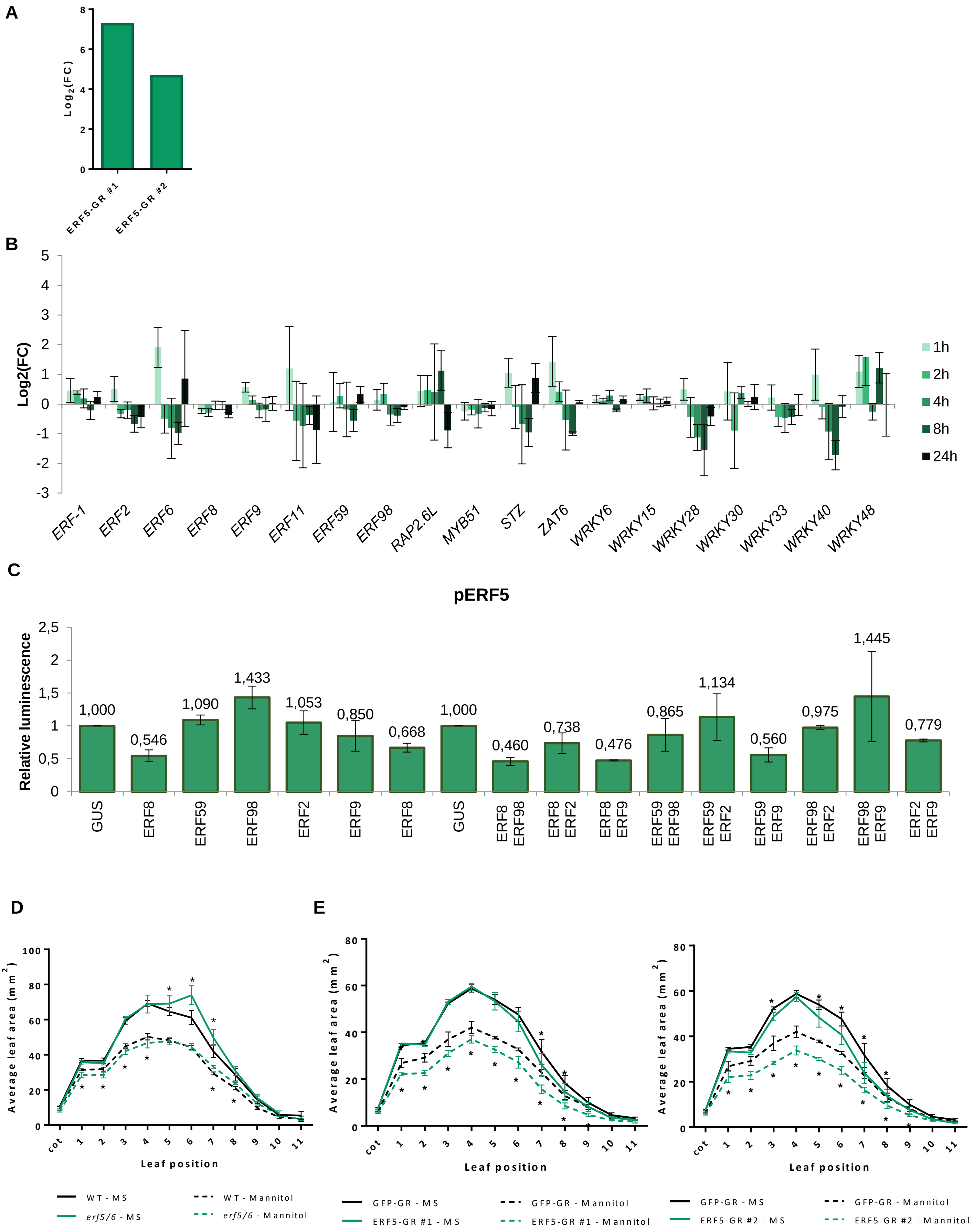

Appendix Figure S5 - Overview of experimental data related to ERF5 (AT5G47230).

A Overexpression level measured in 10-day-old seedlings of two independent inducible overexpression lines.

B The induction of 19 genes encoding transcription factors, 1 h, 2 h, 4 h, 8 h and 24 h after transfer of the inducible overexpression line to dexamethasone-containing medium at 15 DAS.

C Activation of the ERF5 promotor by individual or the combination of two transcription factors with transient expression assays. The presented values are luminescence levels normalized to the negative control, 35S::GUS.

D,E The area of every individual leaf was measured at 22 DAS of *erf5/6* (D) and two independent lines of ERF5-GR (E), on mannitol-containing or control MS medium (supplemented with dexamethasone in case of the inducible overexpression lines).

Data information: data are presented as mean ± SEM. FC = Fold change. n = 1 (A), 3 (B,D,E) independent experiment(s). * = FDR < 0.1 (mixed model analysis, user-defined Wald tests) (B), * = P < 0.05 (mixed model, partial F-tests) (D,E).

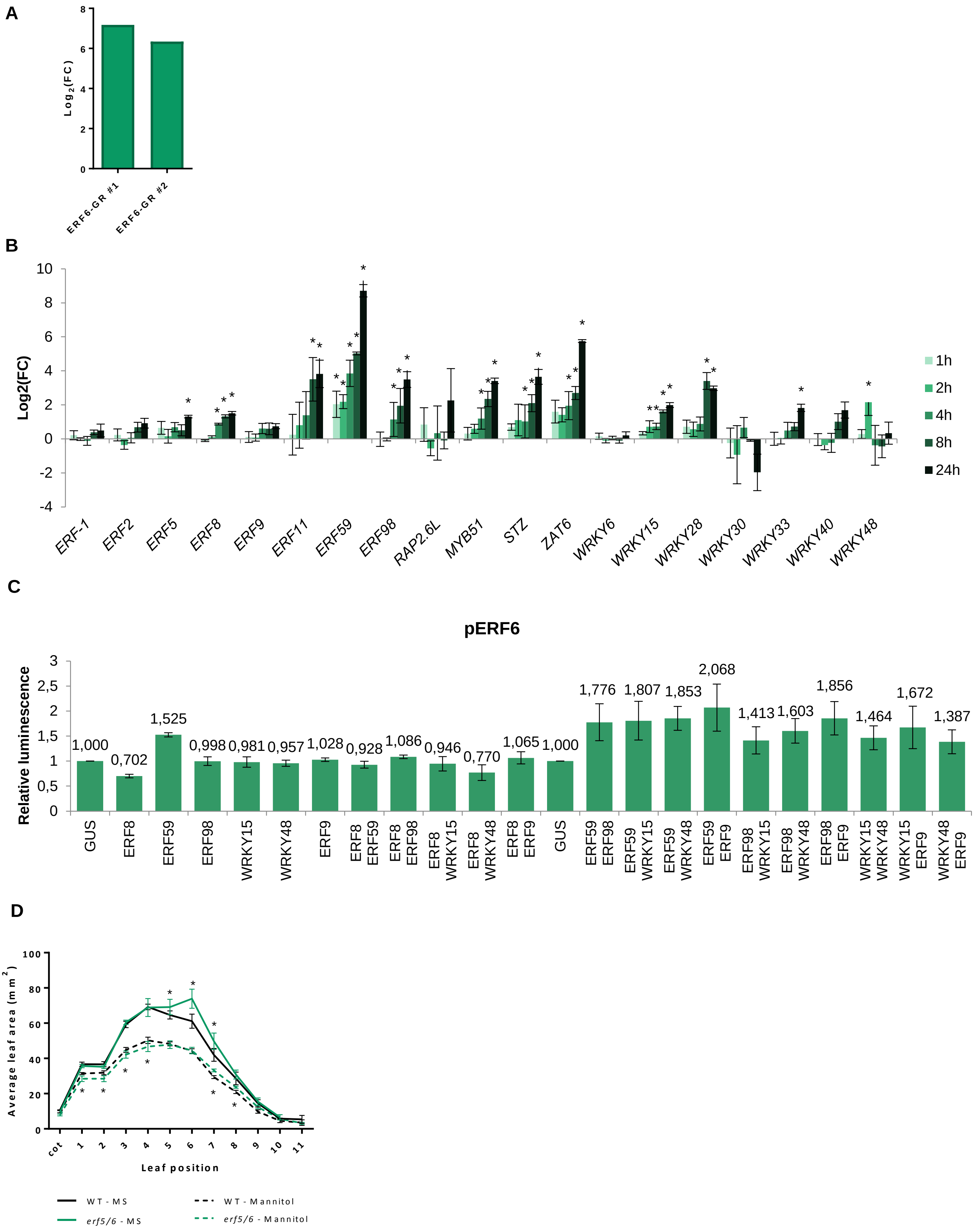

Appendix Figure S6 - Overview of experimental data related to ERF6 (AT4G17490).

A Overexpression level measured in 10-day-old seedlings of two independent inducible overexpression lines.

B The induction of 19 genes encoding transcription factors, 1 h, 2 h, 4 h, 8 h and 24 h after transfer of the inducible overexpression line to dexamethasone-containing medium at 15 DAS.

C Activation of the ERF6 promotor by individual or the combination of two transcription factors with transient expression assays. The presented values are luminescence levels normalized to the negative control, 35S::GUS.

D The area of every individual leaf was measured at 22 DAS of *erf5/6* on mannitol-containing or control MS medium.

Data information: data are presented as mean ± SEM. FC = Fold change. n = 1 (A), 3 (B,D,E) independent experiment(s). * = FDR < 0.1 (mixed model analysis, user-defined Wald tests) (B), * = P < 0.05 (mixed model, partial F-tests) (D).

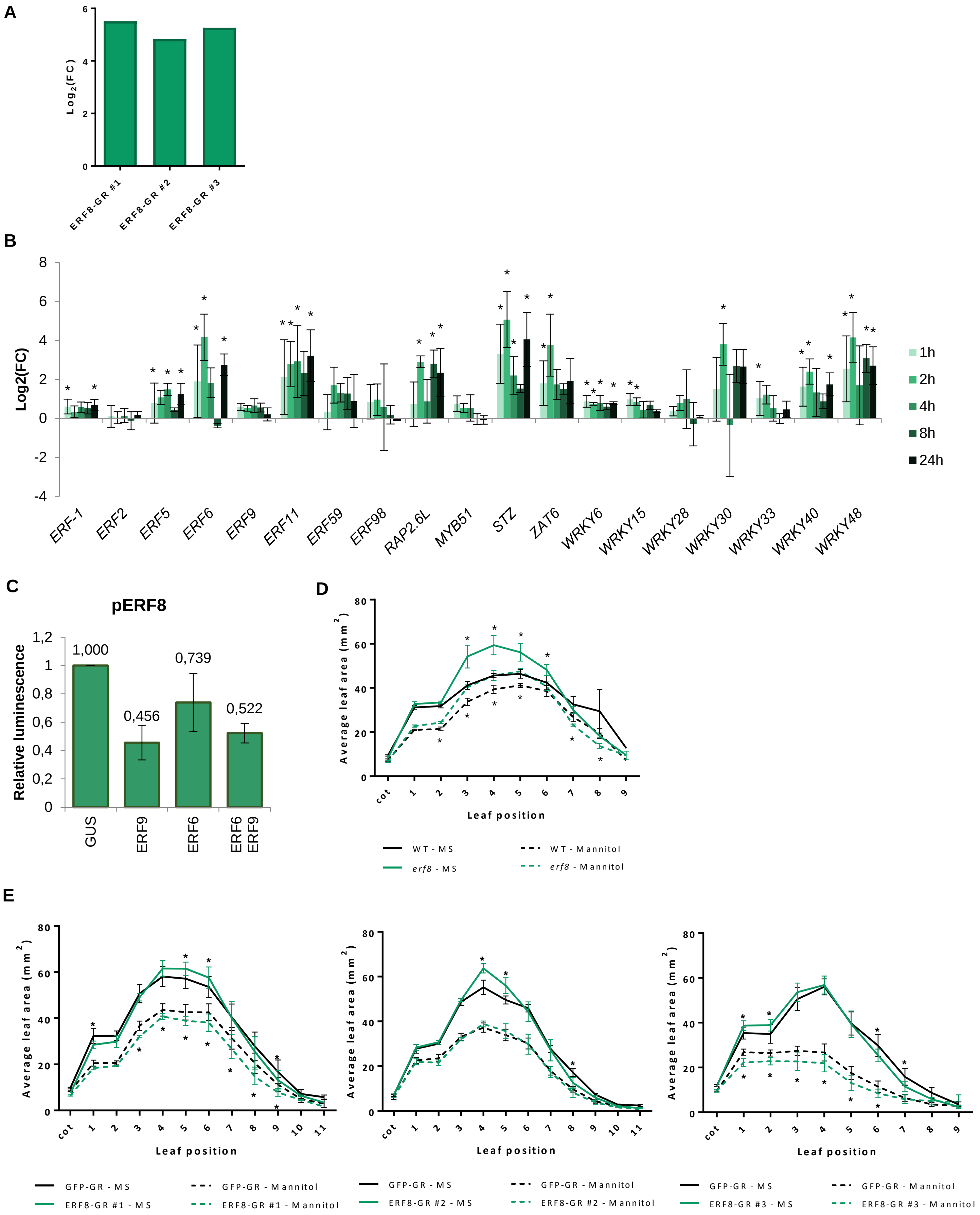

Appendix Figure S7 - Overview of experimental data related to ERF8 (AT1G53170).

A Overexpression level measured in 10-day-old seedlings of three independent inducible overexpression lines.

B The induction of 19 genes encoding transcription factors, 1 h, 2 h, 4 h, 8 h and 24 h after transfer of the inducible overexpression line to dexamethasone-containing medium at 15 DAS.

C Activation of the ERF8 promotor by individual or the combination of two transcription factors with transient expression assays. The presented values are luminescence levels normalized to the negative control, 35S::GUS.

D,E The area of every individual leaf was measured at 22 DAS of *erf8* (D) and three independent lines of ERF8-GR (E), on mannitol-containing or control MS medium (supplemented with dexamethasone in case of the inducible overexpression lines).

Data information: data are presented as mean ± SEM. FC = Fold change. n = 1 (A), 3 (B,D,E) independent experiment(s). * = FDR < 0.1 (mixed model analysis, user-defined Wald tests) (B), * = P < 0.05 (mixed model, partial F-tests) (D,E).

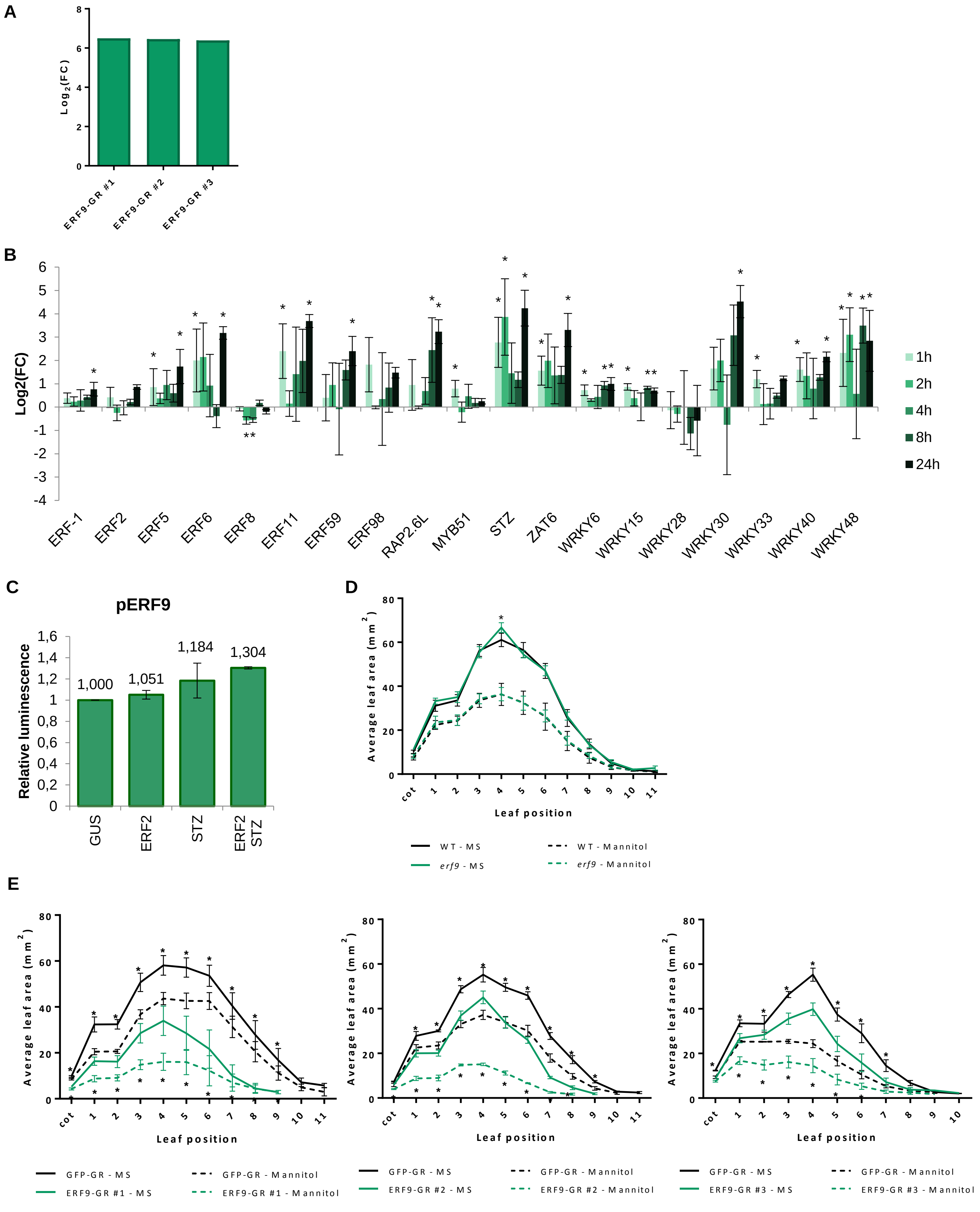

Appendix Figure S8 - Overview of experimental data related to ERF9 (AT5G44210).

A Overexpression level measured in 10-day-old seedlings of three independent inducible overexpression lines.

B The induction of 19 genes encoding transcription factors, 1 h, 2 h, 4 h, 8 h and 24 h after transfer of the inducible overexpression line to dexamethasone-containing medium at 15 DAS.

C Activation of the ERF9 promotor by individual or the combination of two transcription factors with transient expression assays. The presented values are luminescence levels normalized to the negative control, 35S::GUS.

D,E The area of every individual leaf was measured at 22 DAS of *erf9* (D) and three independent lines of ERF9-GR (E), on mannitol-containing or control MS medium (supplemented with dexamethasone in case of the inducible overexpression lines).

Data information: data are presented as mean ± SEM. FC = Fold change. n = 1 (A), 3 (B,D,E) independent experiment(s). * = FDR < 0.1 (mixed model analysis, user-defined Wald tests) (B), * = P < 0.05 (mixed model, partial F-tests) (D,E).

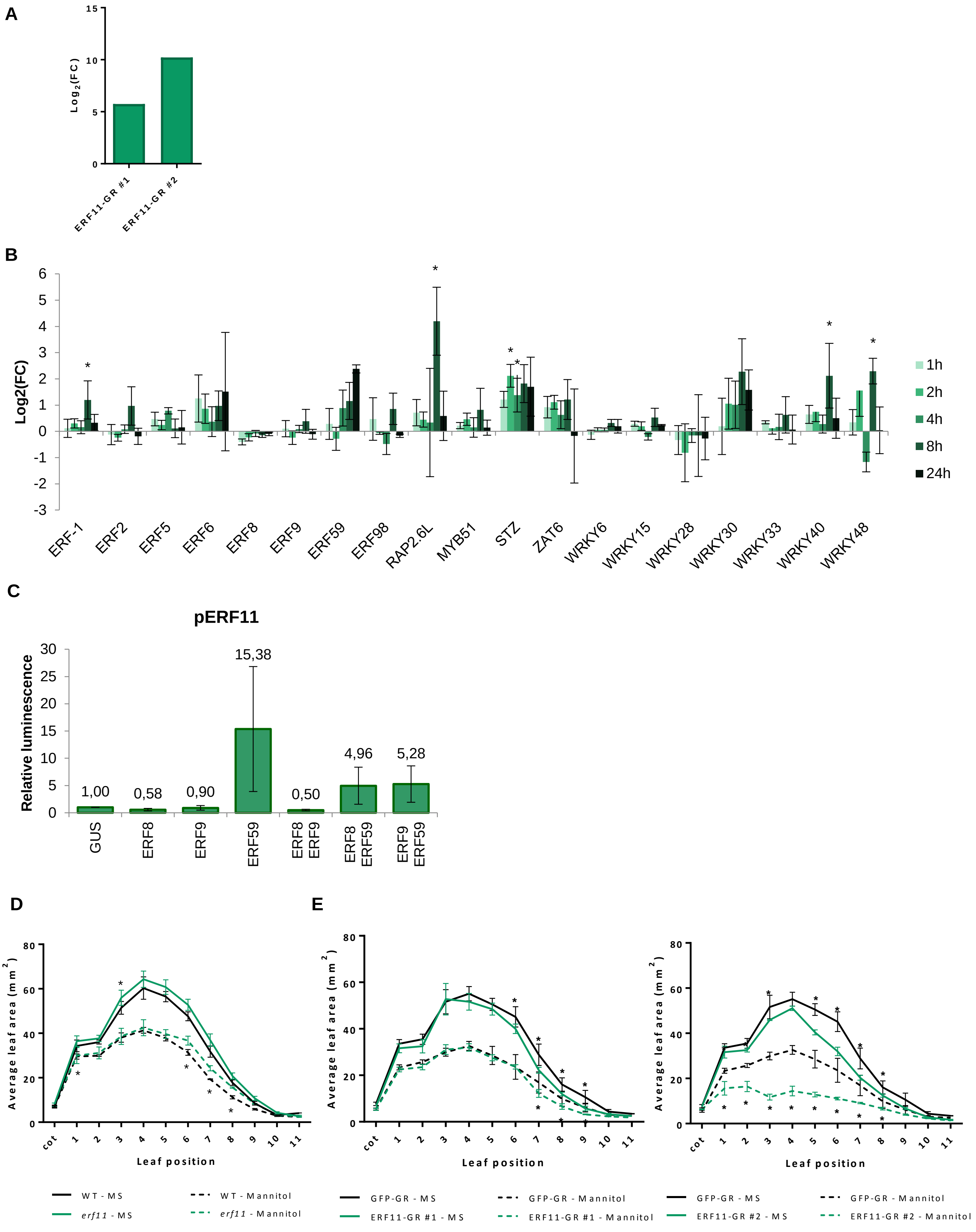

Appendix Figure S9 - Overview of experimental data related to ERF11 (AT1G28370).

A Overexpression level measured in 10-day-old seedlings of two independent inducible overexpression lines.

B The induction of 19 genes encoding transcription factors, 1 h, 2 h, 4 h, 8 h and 24 h after transfer of the inducible overexpression line to dexamethasone-containing medium at 15 DAS.

C Activation of the ERF11 promotor by individual or the combination of two transcription factors with transient expression assays. The presented values are luminescence levels normalized to the negative control, 35S::GUS.

D,E The area of every individual leaf was measured at 22 DAS of *erf11* (D) and two independent lines of ERF11-GR (E), on mannitol-containing or control MS medium (supplemented with dexamethasone in case of the inducible overexpression lines).

Data information: data are presented as mean ± SEM. FC = Fold change. n = 1 (A), 3 (B,D,E) independent experiment(s). * = FDR < 0.1 (mixed model analysis, user-defined Wald tests) (B), * = P < 0.05 (mixed model, partial F-tests) (D,E).

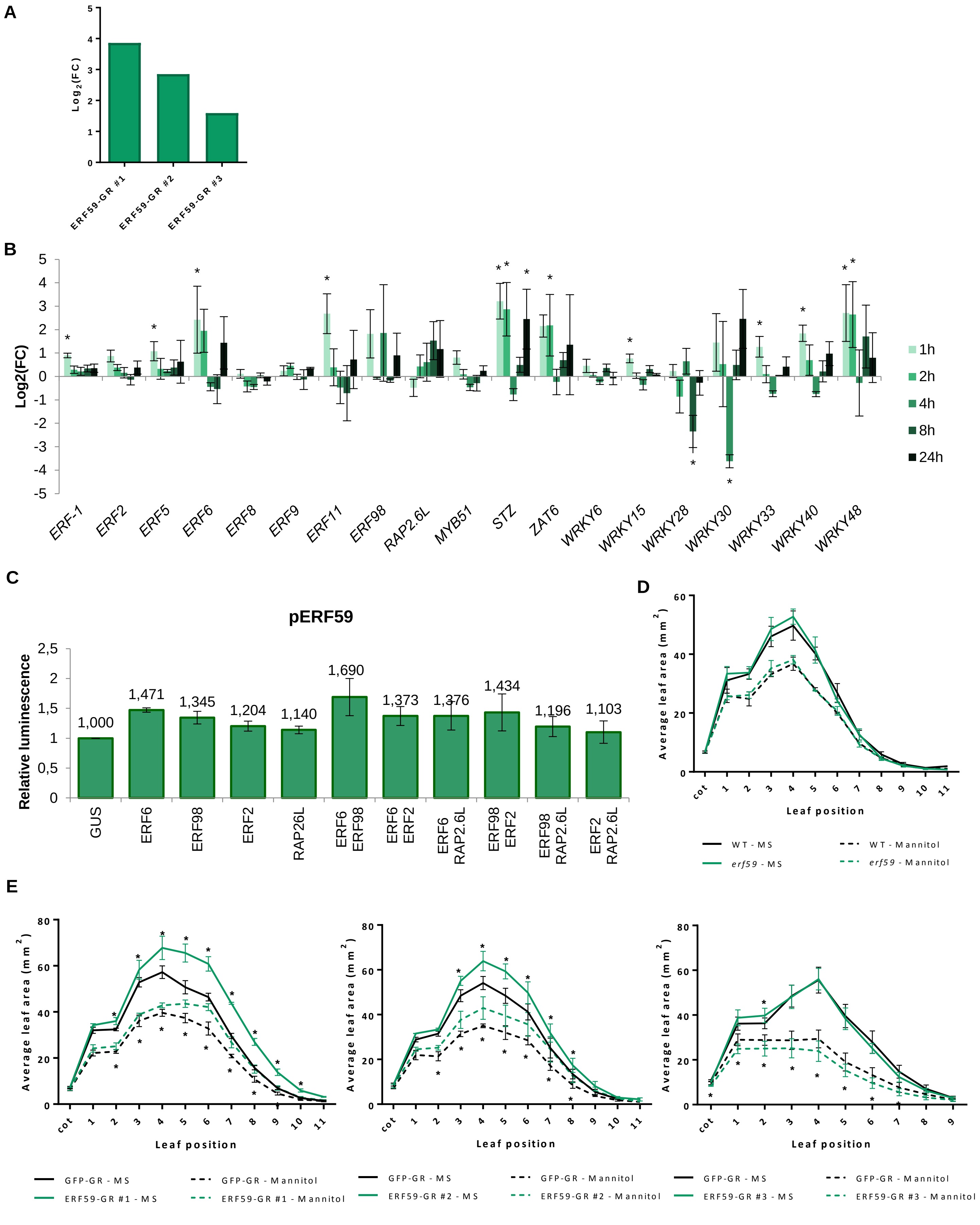

Appendix Figure S10 - Overview of experimental data related to ERF59 (AT1G06160).
A Overexpression level measured in 10-day-old seedlings of three independent inducible overexpression lines.
B The induction of 19 genes encoding transcription factors, 1 h, 2 h, 4 h, 8 h and 24 h after transfer of the inducible overexpression line to dexamethasone-containing medium at 15 DAS.
C Activation of the ERF59 promotor by individual or the combination of two transcription factors with transient expression assays. The presented values are luminescence levels normalized to the negative control, 35S::GUS.
D,E The area of every individual leaf was measured at 22 DAS of *erf59* (D) and three independent lines of ERF59-GR (E), on mannitol-containing or control MS medium (supplemented with dexamethasone in case of the inducible overexpression lines).
Data information: data are presented as mean ± SEM. FC = Fold change. n = 1 (A), 3 (B,D,E) independent experiment(s). * = FDR < 0.1 (mixed model analysis, user-defined Wald tests) (B), * = P < 0.05 (mixed model, partial F-tests) (D,E).

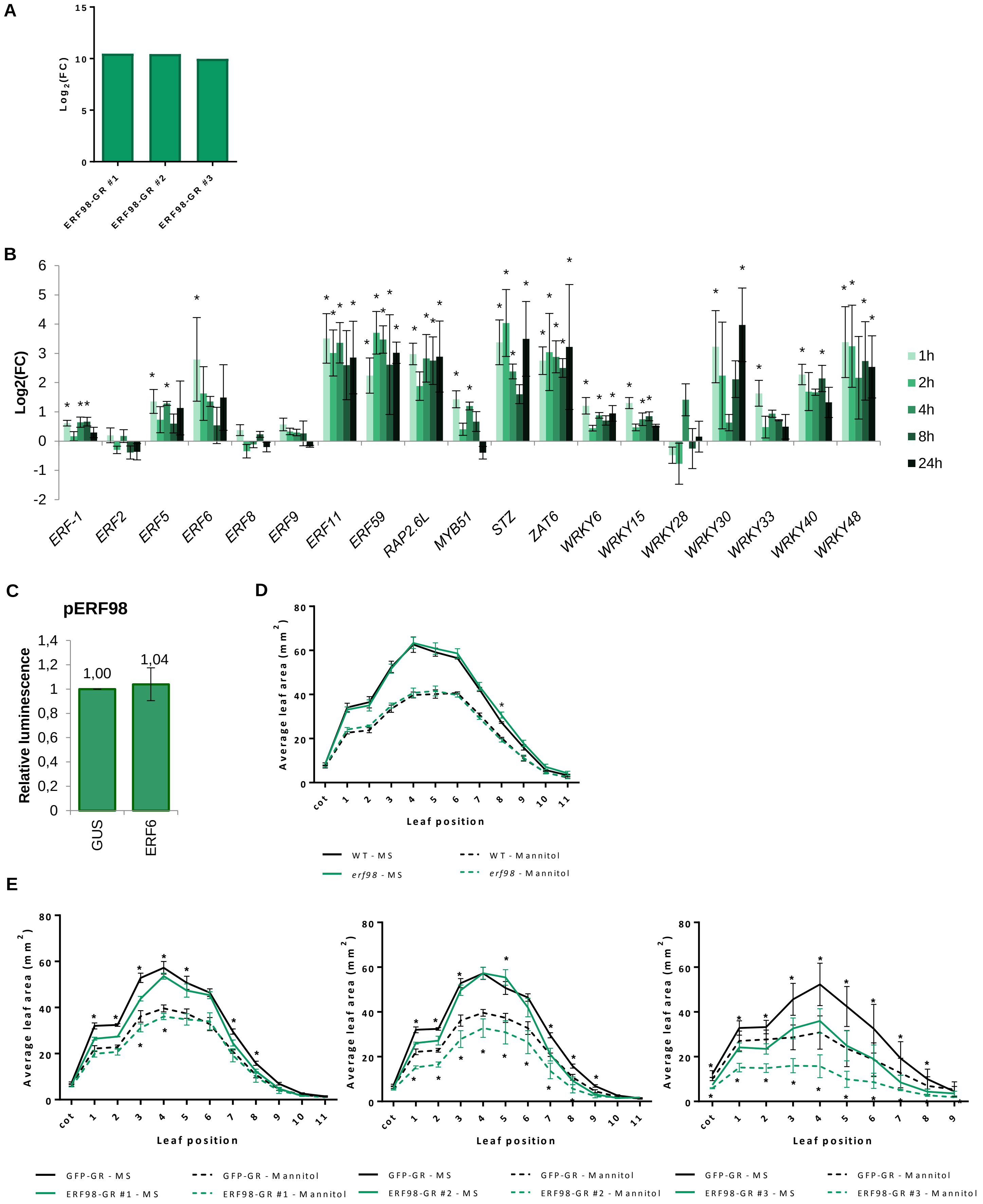

Appendix Figure S11 - Overview of experimental data related to ERF98 (AT3G23230).

A Overexpression level measured in 10-day-old seedlings of three independent inducible overexpression lines.

B The induction of 19 genes encoding transcription factors, 1 h, 2 h, 4 h, 8 h and 24 h after transfer of the inducible overexpression line to dexamethasone-containing medium at 15 DAS.

C Activation of the ERF98 promotor by individual or the combination of two transcription factors with transient expression assays. The presented values are luminescence levels normalized to the negative control, 35S::GUS.

D,E The area of every individual leaf was measured at 22 DAS of *erf98* (D) and three independent lines of ERF98-GR (E), on mannitol-containing or control MS medium (supplemented with dexamethasone in case of the inducible overexpression lines).

Data information: data are presented as mean ± SEM. FC = Fold change. n = 1 (A), 3 (B,D,E) independent experiment(s). * = FDR < 0.1 (mixed model analysis, user-defined Wald tests) (B), * = P < 0.05 (mixed model, partial F-tests) (D,E).

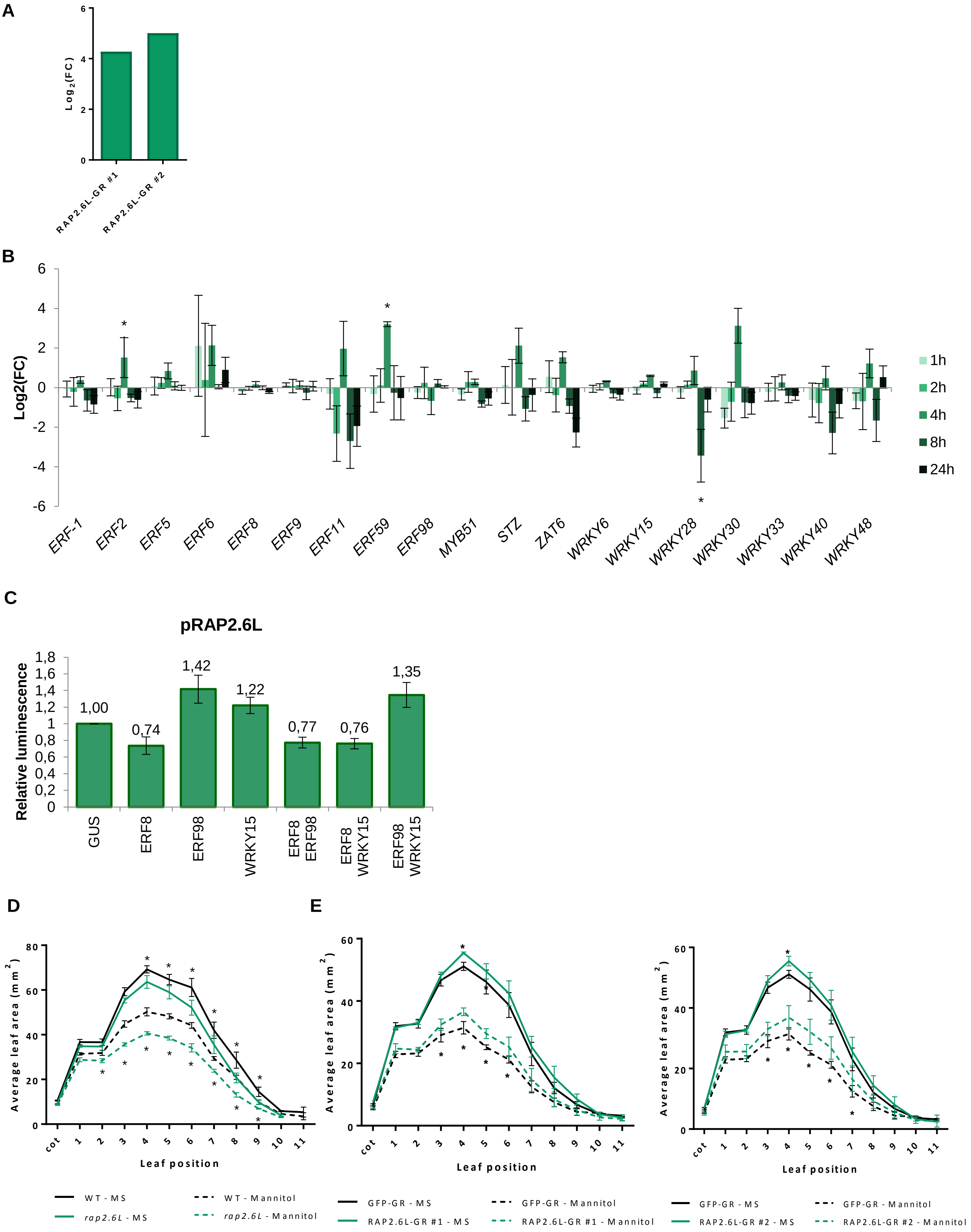

Appendix Figure S12 - Overview of experimental data related to RAP2.6L (AT5G13330).

A Overexpression level measured in 10-day-old seedlings of two independent inducible overexpression lines.

B The induction of 19 genes encoding transcription factors, 1 h, 2 h, 4 h, 8 h and 24 h after transfer of the inducible overexpression line to dexamethasone-containing medium at 15 DAS.

C Activation of the RAP2.6L promotor by individual or the combination of two transcription factors with transient expression assays. The presented values are luminescence levels normalized to the negative control, 35S::GUS.

D,E The area of every individual leaf was measured at 22 DAS of *rap2.6L*(D) and two independent lines of RAP2.6L-GR (E), on mannitol-containing or control MS medium (supplemented with dexamethasone in case of the inducible overexpression lines).

Data information: data are presented as mean ± SEM. FC = Fold change. n = 1 (A), 3 (B,D,E) independent experiment(s). * = FDR < 0.1 (mixed model analysis, user-defined Wald tests) (B), * = P < 0.05 (mixed model, partial F-tests) (D,E).

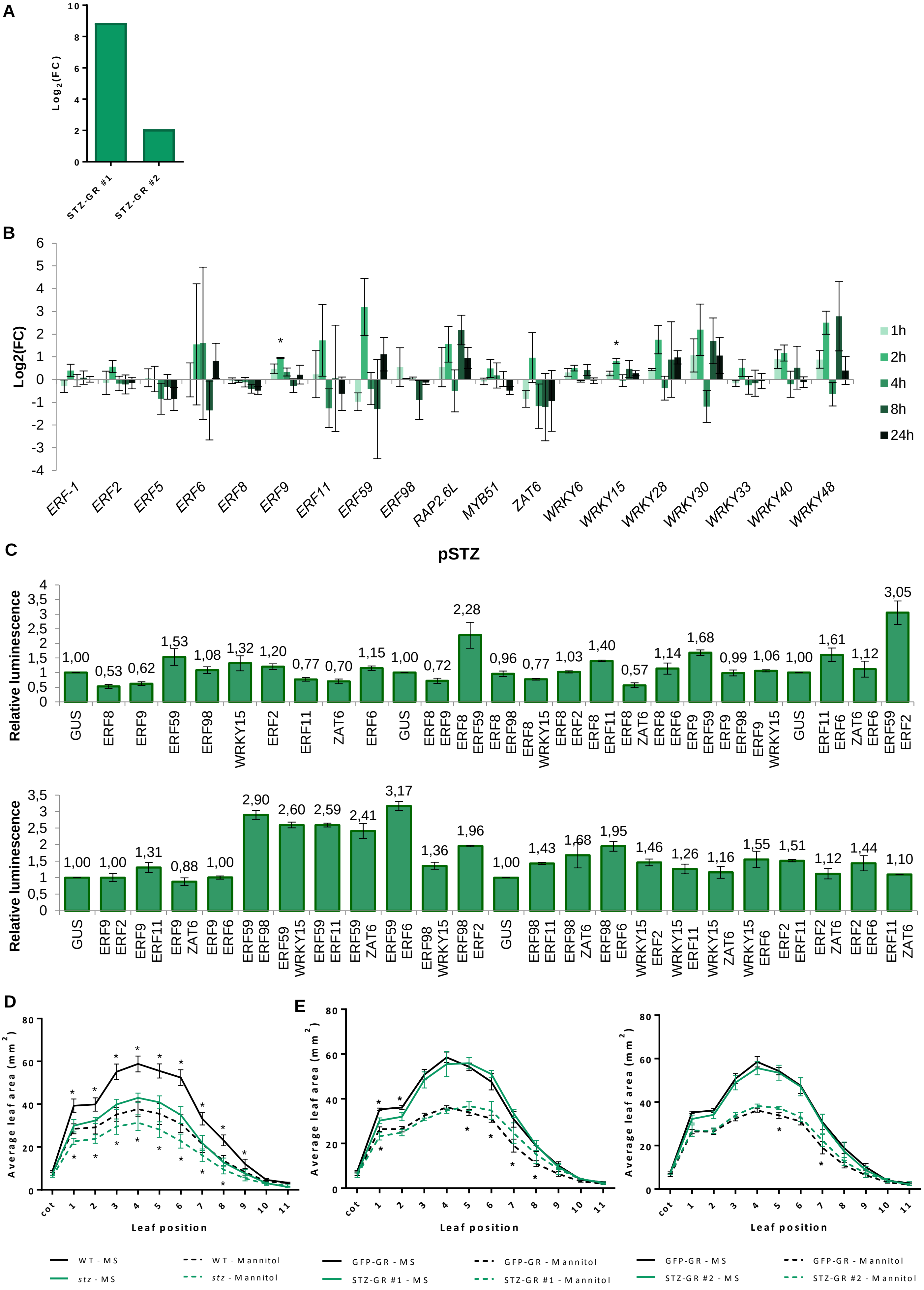

Appendix Figure S13 - Overview of experimental data related to STZ (AT1G27730).

A Overexpression level measured in 10-day-old seedlings of two independent inducible overexpression lines.

B The induction of 19 genes encoding transcription factors, 1 h, 2 h, 4 h, 8 h and 24 h after transfer of the inducible overexpression line to dexamethasone-containing medium at 15 DAS.

C Activation of the STZ promotor by individual or the combination of two transcription factors with transient expression assays. The presented values are luminescence levels normalized to the negative control, 35S::GUS.

D,E The area of every individual leaf was measured at 22 DAS of *stz* (D) and two independent lines of STZ-GR (E), on mannitol-containing or control MS medium (supplemented with dexamethasone in case of the inducible overexpression lines).

Data information: data are presented as mean ± SEM. FC = Fold change. n = 1 (A), 3 (B,D,E) independent experiment(s). * = FDR < 0.1 (mixed model analysis, user-defined Wald tests) (B), * = P < 0.05 (mixed model, partial F-tests) (D,E).

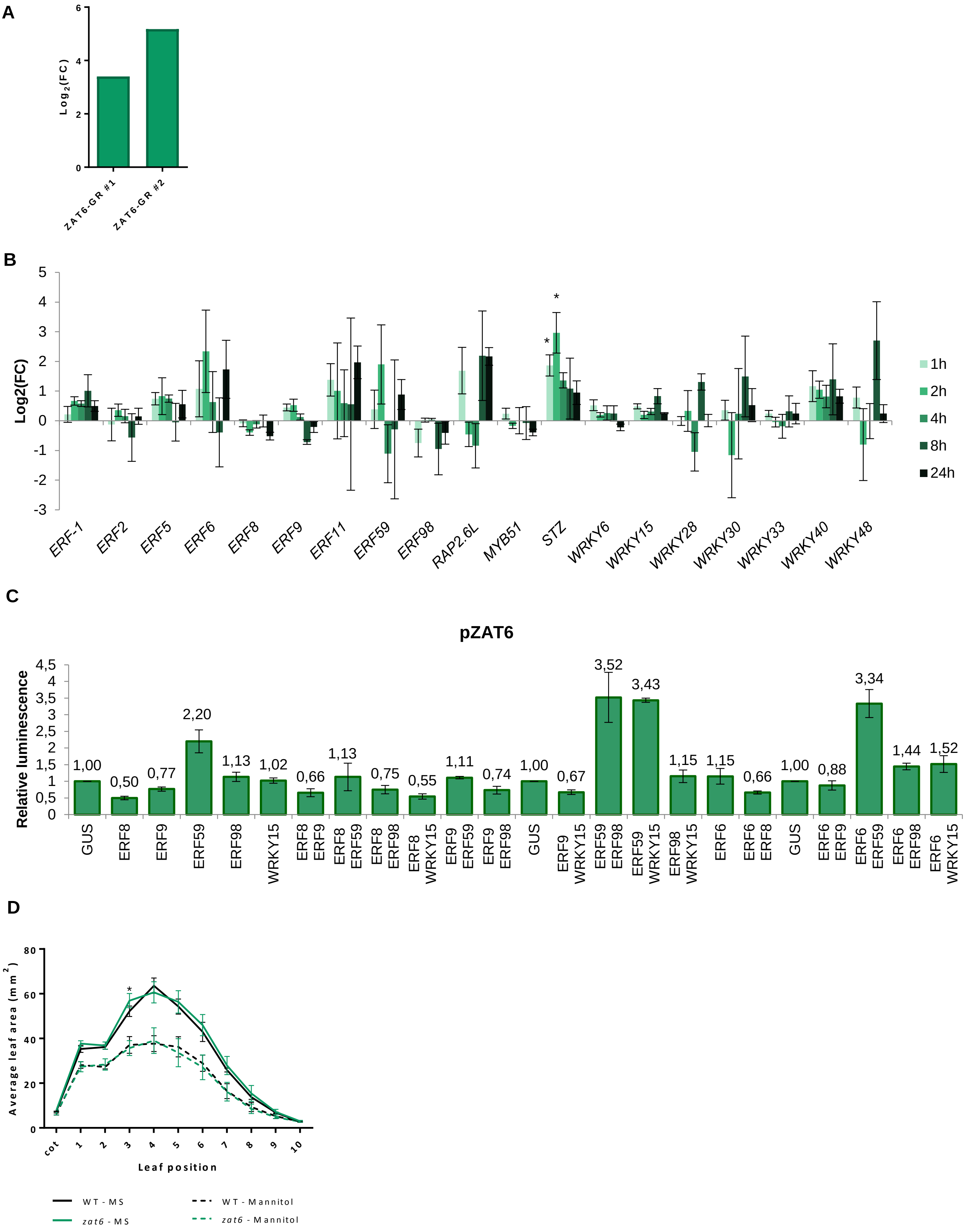

Appendix Figure S14 - Overview of experimental data related to ZAT6 (AT5G04340).

A Overexpression level measured in 10-day-old seedlings of two independent inducible overexpression lines.

B The induction of 19 genes encoding transcription factors, 1 h, 2 h, 4 h, 8 h and 24 h after transfer of the inducible overexpression line to dexamethasone-containing medium at 15 DAS.

C Activation of the ZAT6 promotor by individual or the combination of two transcription factors with transient expression assays. The presented values are luminescence levels normalized to the negative control, 35S::GUS.

D The area of every individual leaf was measured at 22 DAS of *zat6* on mannitol-containing or control MS medium.

Data information: data are presented as mean ± SEM. FC = Fold change. n = 1 (A), 3 (B,D,E) independent experiment(s). * = FDR < 0.1 (mixed model analysis, user-defined Wald tests) (B), * = P < 0.05 (mixed model, partial F-tests) (D).

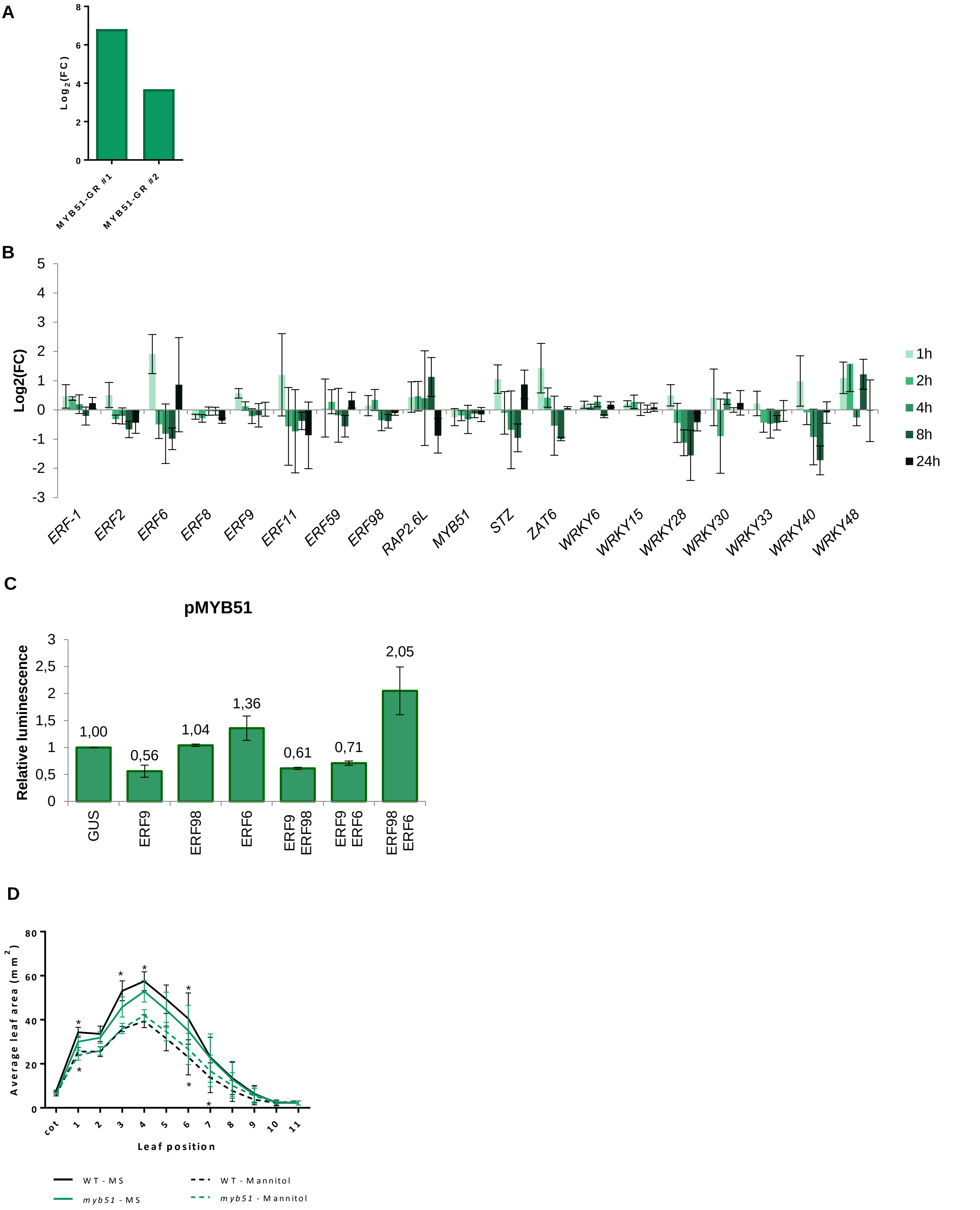

Appendix Figure S15 - Overview of experimental data related to MYB51 (AT1G18570).

A Overexpression level measured in 10-day-old seedlings of two independent inducible overexpression lines.

B The induction of 19 genes encoding transcription factors, 1 h, 2 h, 4 h, 8 h and 24 h after transfer of the inducible overexpression line to dexamethasone-containing medium at 15 DAS.

C Activation of the MYB51 promotor by individual or the combination of two transcription factors with transient expression assays. The presented values are luminescence levels normalized to the negative control, 35S::GUS.

D The area of every individual leaf was measured at 22 DAS of *myb51* on mannitol-containing or control MS medium.

Data information: data are presented as mean ± SEM. FC = Fold change. n = 1 (A), 3 (B,D,E) independent experiment(s). * = FDR < 0.1 (mixed model analysis, user-defined Wald tests) (B), * = P < 0.05 (mixed model, partial F-tests) (D).

A

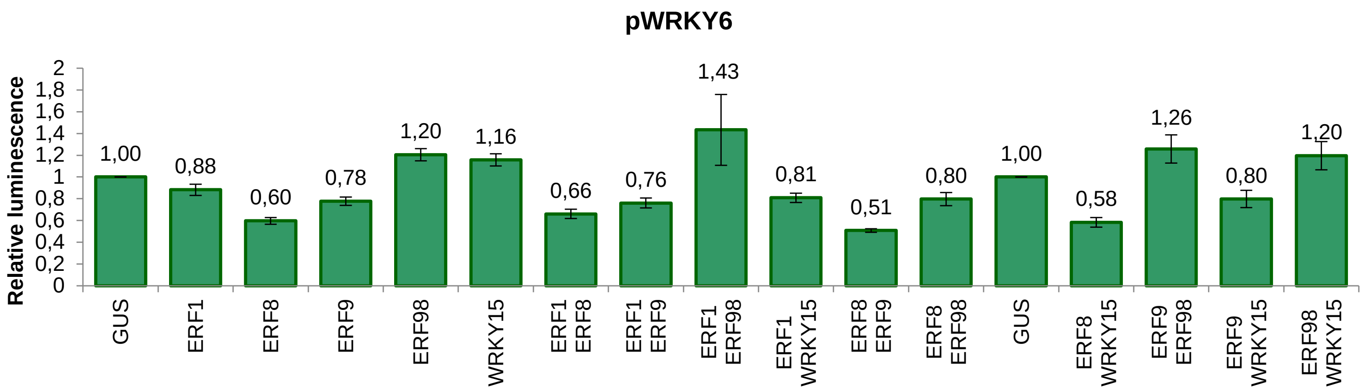

B

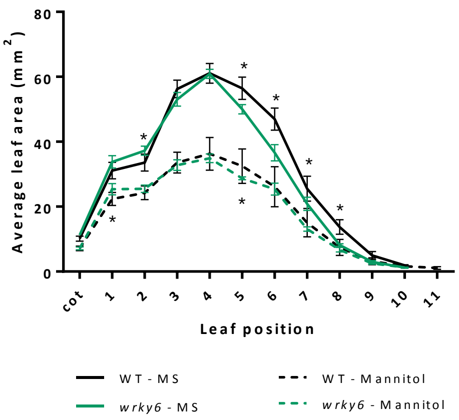

Appendix Figure S16 - Overview of experimental data related to WRKY6 (AT1G62300).

A Activation of the WRKY6 promotor by individual or the combination of two transcription factors with transient expression assays. The presented values are luminescence levels normalized to the negative control, 35S::GUS.

B The area of every individual leaf was measured at 22 DAS of *wrky6* on mannitol-containing or control MS medium.

Data information: data are presented as mean ± SEM. n = 3 independent experiments. * = P < 0.05 (mixed model, partial F-tests) (B).

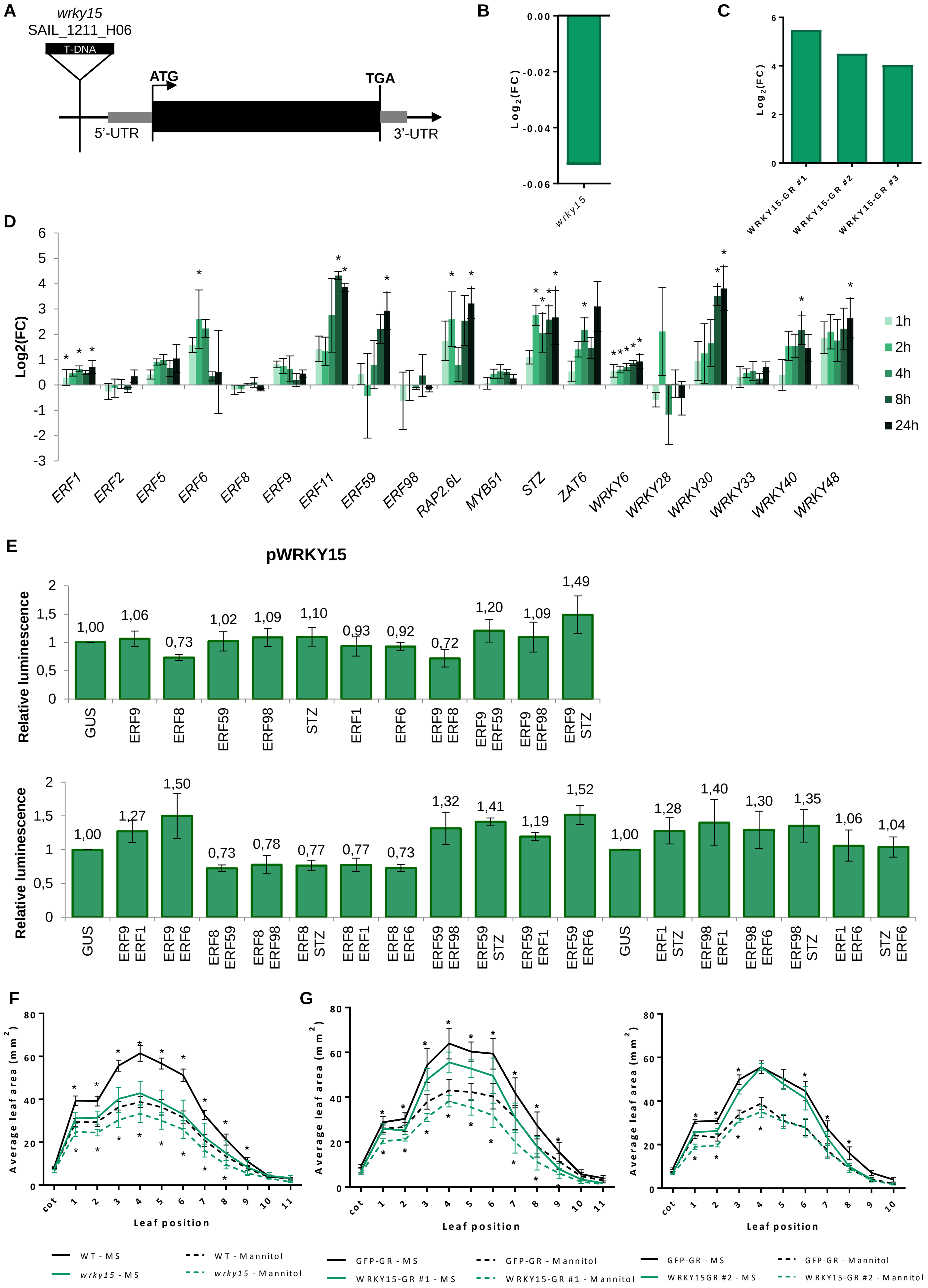

Appendix Figure S17 - Overview of experimental data related to WRKY15 (AT2G23320).

A Schematic representation of the gene with the position of the T-DNA insertion. Start and stop codon, 3'-UTR and 5'-UTR are indicated.

B,C Knock-down and overexpression measured in 10-day-old seedlings of a T-DNA insertion line (B) and three independent inducible overexpression lines (C).

D The induction of 19 genes encoding transcription factors, 1 h, 2 h, 4 h, 8 h and 24 h after transfer of the inducible overexpression line to dexamethasone-containing medium at 15 DAS.

E Activation of the WRKY15 promotor by individual or the combination of two transcription factors with transient expression assays. The presented values are luminescence levels normalized to the negative control, 35S::GUS.

F,G The area of every individual leaf was measured at 22 DAS of *wrky15* (F) and two independent lines of WRKY15-GR (G), on mannitol-containing or control MS medium (supplemented with dexamethasone in case of the inducible overexpression lines).

Data information: data are presented as mean ± SEM. FC = Fold change. n = 1 (B,C), 3 (D,E,F,G) independent experiment(s). * = FDR < 0.1 (mixed model analysis, user-defined Wald tests) (D), * = P < 0.05 (mixed model, partial F-tests) (F,G).

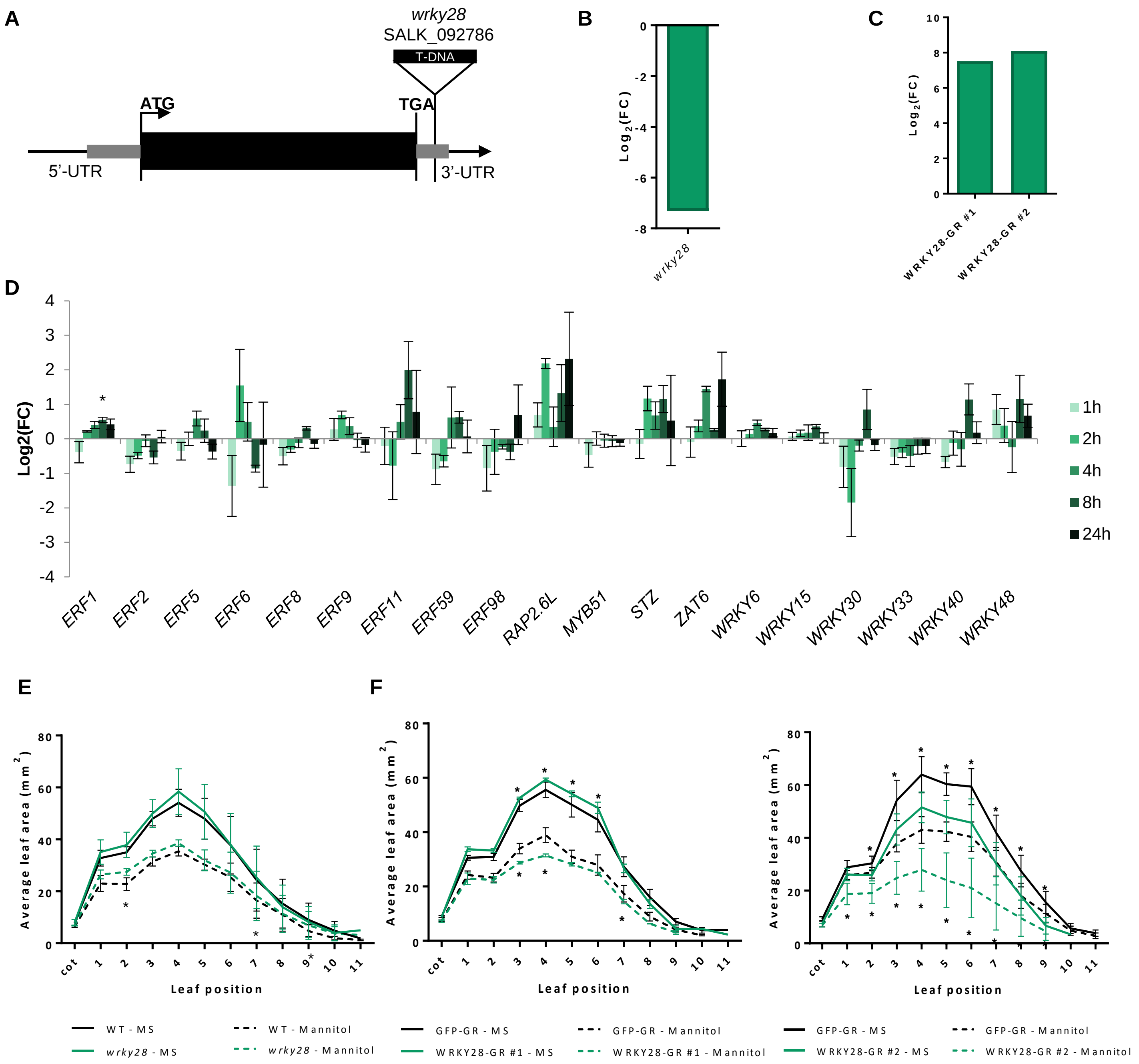

Appendix Figure S18 - Overview of experimental data related to WRKY28 (AT4G18170).

A Schematic representation of the gene with the position of the T-DNA insertion. Start and stop codon, 3'-UTR and 5'-UTR are indicated.

B,C Knock-down and overexpression measured in 10-day-old seedlings of a T-DNA insertion line (B) and two independent inducible overexpression lines (C).

D The induction of 19 genes encoding transcription factors, 1 h, 2 h, 4 h, 8 h and 24 h after transfer of the inducible overexpression line to dexamethasone-containing medium at 15 DAS.

E,F The area of every individual leaf was measured at 22 DAS of *wrky28* (E) and two independent lines of WRKY28-GR (F), on mannitol-containing or control MS medium (supplemented with dexamethasone in case of the inducible overexpression lines).

Data information: data are presented as mean ± SEM. FC = Fold change. n = 1 (B,C), 3 (D,E,F) independent experiment(s). * = FDR < 0.1 (mixed model analysis, user-defined Wald tests) (D), * = P < 0.05 (mixed model, partial F-tests) (E,F).

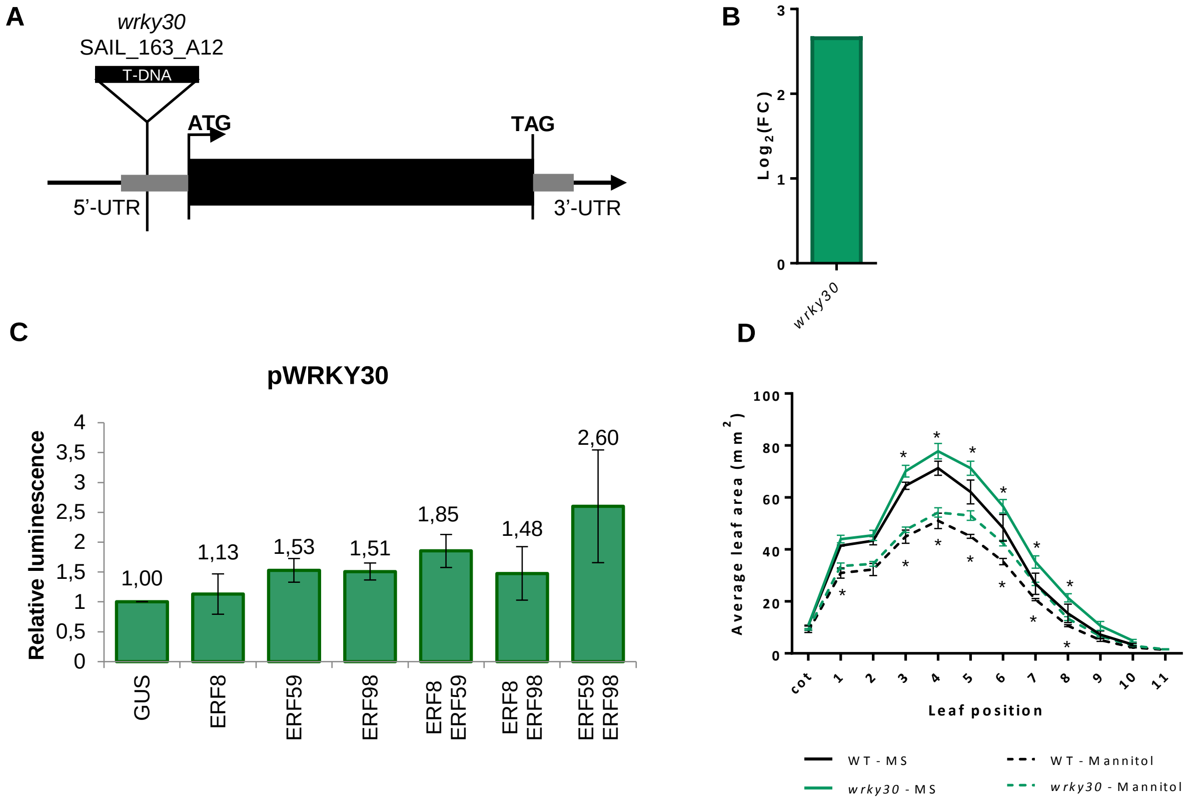

Appendix Figure S19 - Overview of experimental data related to WRKY30 (AT5G24110).

A Schematic representation of the gene with the position of the T-DNA insertion. Start and stop codon, 3'-UTR and 5'-UTR are indicated.

B Knock-down in 10-day-old seedlings of a T-DNA insertion line.

C Activation of the WRKY30 promotor by individual or the combination of two transcription factors with transient expression assays. The presented values are luminescence levels normalized to the negative control, 35S::GUS.

D The area of every individual leaf was measured at 22 DAS of *wrky30* on mannitol-containing or control MS medium.

Data information: data are presented as mean ± SEM. FC = Fold change. n = 1 (B), 3 (C,D) independent experiment(s). * = $P < 0.05$ (mixed model, partial F-tests) (D).

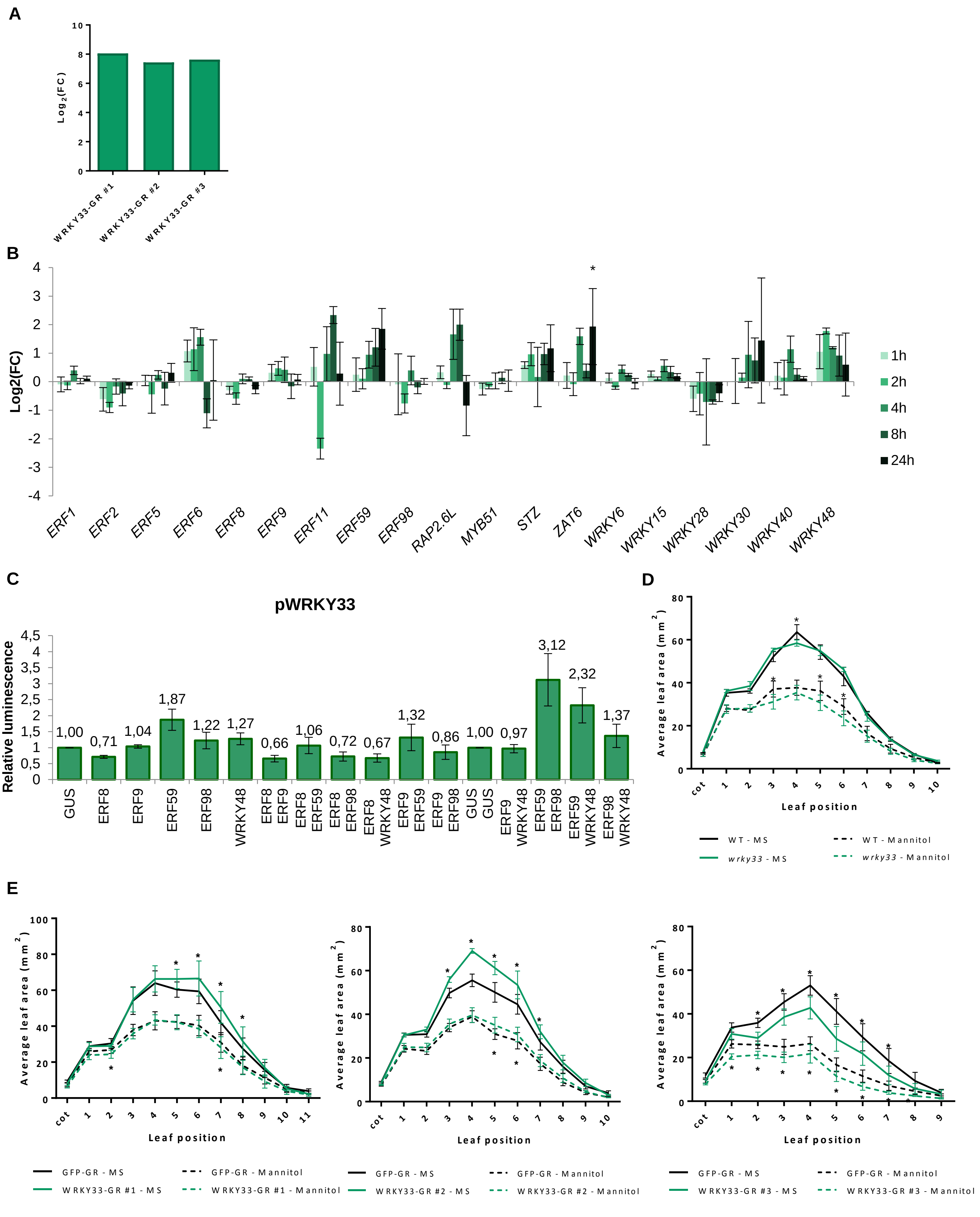

Appendix Figure S20 - Overview of experimental data related to WRKY33.

A Overexpression level measured in 8- or 9-day-old seedlings of three independent inducible overexpression lines.

B The induction of 19 genes encoding transcription factors, 1 h, 2 h, 4 h, 8 h and 24 h after transfer of the inducible overexpression line to dexamethasone-containing medium at 15 DAS.

C Activation of the WRKY33 promotor by individual or the combination of two transcription factors with transient expression assays. The presented values are luminescence levels normalized to the negative control, 35S::GUS.

D,E The area of every individual leaf was measured at 22 DAS of *wrky33* (D) and three independent lines of WRKY33-GR (E), on mannitol-containing or control MS medium (supplemented with dexamethasone in case of the inducible overexpression lines).

Data information: data are presented as mean ± SEM. FC = Fold change. n = 1 (A), 3 (B,D,E) independent experiment(s). * = FDR < 0.1 (mixed model analysis, user-defined Wald tests) (B), * = P < 0.05 (mixed model, partial F-tests) (D,E).

A

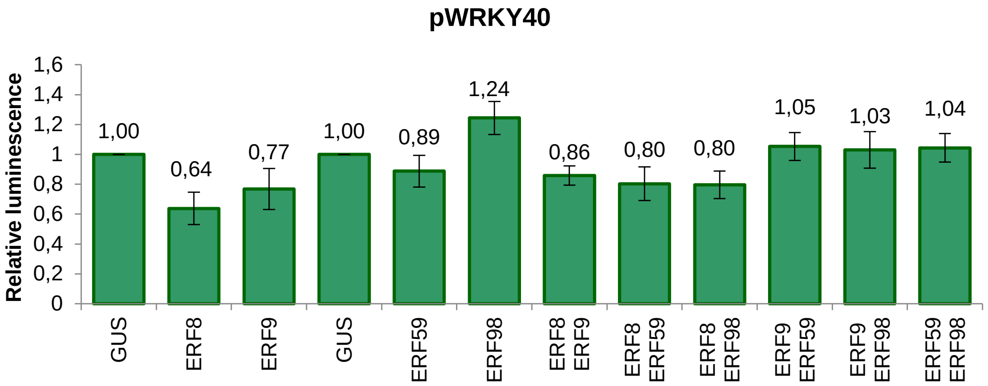

B

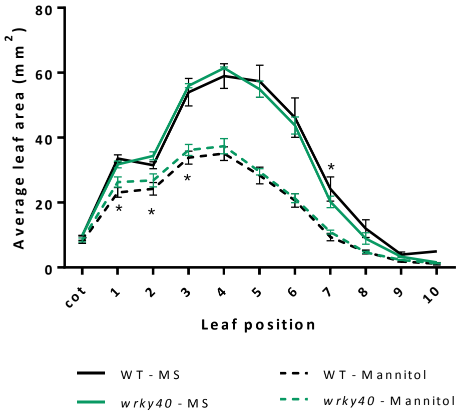

Appendix Figure S21 - Overview of experimental data related to WRKY40 (AT1G80840).

A Activation of the WRKY40 promotor by individual or the combination of two transcription factors with transient expression assays. The presented values are luminescence levels normalized to the negative control, 35S::GUS.

B The area of every individual leaf was measured at 22 DAS of *wrky40* on mannitol-containing or control MS medium.

Data information: data are presented as mean ± SEM. n = 3 independent experiments. * = P < 0.05 (mixed model, partial F-tests) (B).

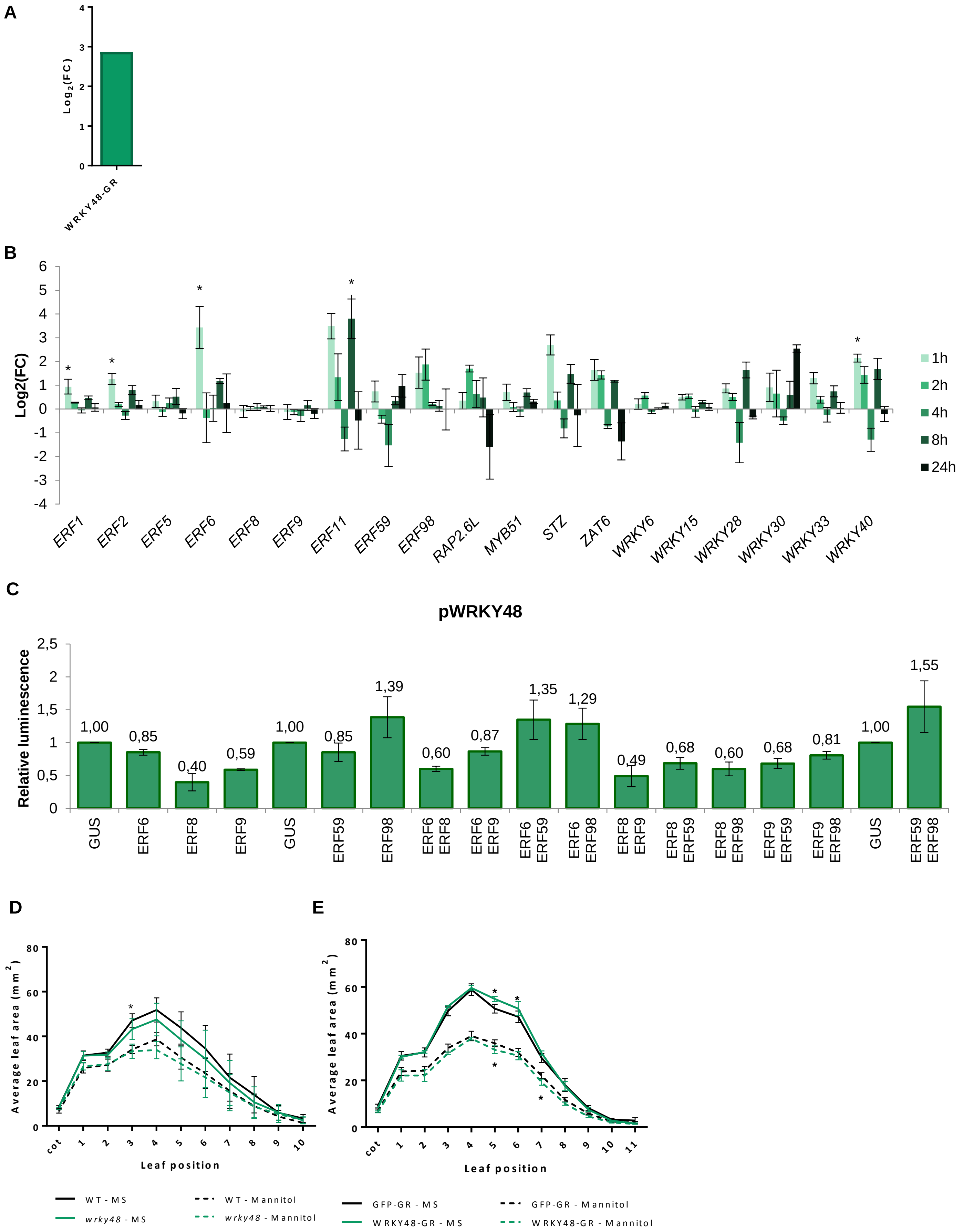

Appendix Figure S22 - Overview of experimental data related to WRKY48 (AT5G49520).

A Overexpression level measured in 8- or 9-day-old seedlings of inducible overexpression lines.

B The induction of 19 genes encoding transcription factors, 1 h, 2 h, 4 h, 8 h and 24 h after transfer of the inducible overexpression line to dexamethasone-containing medium at 15 DAS.

C Activation of the WRKY48 promotor by individual or the combination of two transcription factors with transient expression assays. The presented values are luminescence levels normalized to the negative control, 35S::GUS.

D,E The area of every individual leaf was measured at 22 DAS of *wrky48* (D) and two independent lines of WRKY48-GR (E), on mannitol-containing or control MS medium (supplemented with dexamethasone in case of the inducible overexpression lines).

Data information: data are presented as mean ± SEM. FC = Fold change. n = 1 (A), 3 (B,D,E) independent experiment(s). * = FDR < 0.1 (mixed model analysis, user-defined Wald tests) (B), * = P < 0.05 (mixed model, partial F-tests) (D,E).

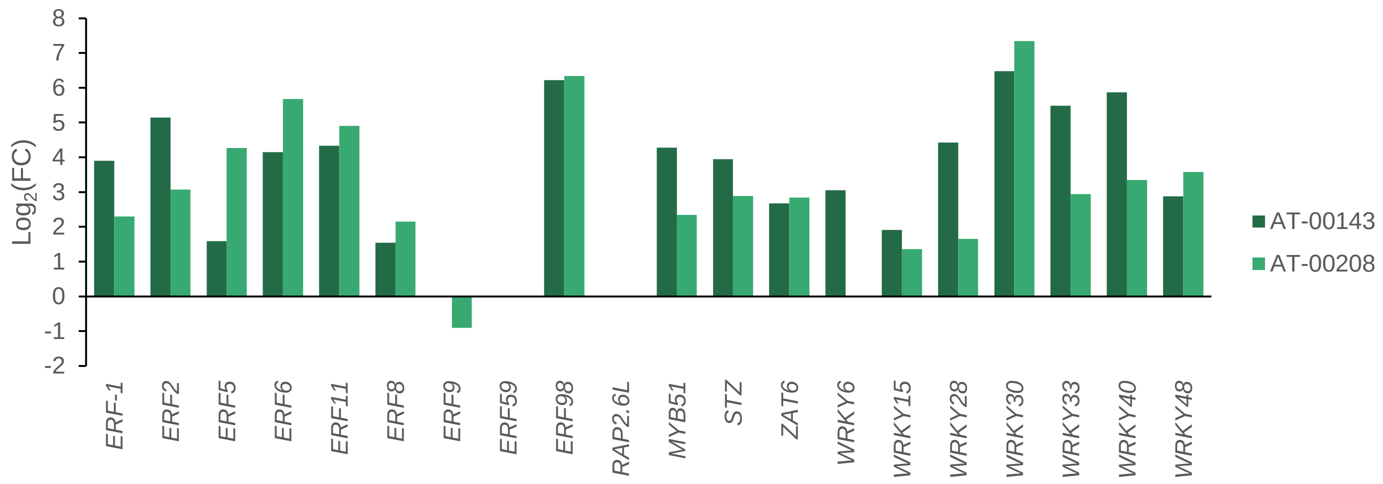

Appendix Figure S23 – The differential expression of 20 transcription factors upon cycloheximide treatment.

With the "differential expression" tool in Genevestigator®, the log2(fold change [FC], FDR<0.05) was calculated of the 20 transcription factors upon cycloheximide treatment in two experiments (AT-00143 and AT-00208)) (Hruz et al., 2008).

Appendix Table S2 – Overview of the GOF lines used for the large-scale expression analysis and the crosses.

A large-scale expression analysis was performed in which putative direct and indirect targets were identified. The independent GOF lines used for this analysis and used for the crosses is presented in the table. NA = Not Applicable.

Gene	GOF line used for expression analysis	GOF line used for cross
ERF-1	#2	
ERF2	#1	
ERF5	#1	
ERF6	#1	#1
ERF8	#2	#2
ERF9	#2	#2
ERF11	#2	
ERF59	#1	#1
ERF98	#1	#1
RAP2.6L	#1	
STZ	#1	
ZAT6	#1	
MYB51	#1	
WRKY6	NA	
WRKY15	#2	
WRKY28	#2	
WRKY30	NA	
WRKY33	#2	
WRKY40	NA	
WRKY48	#1	

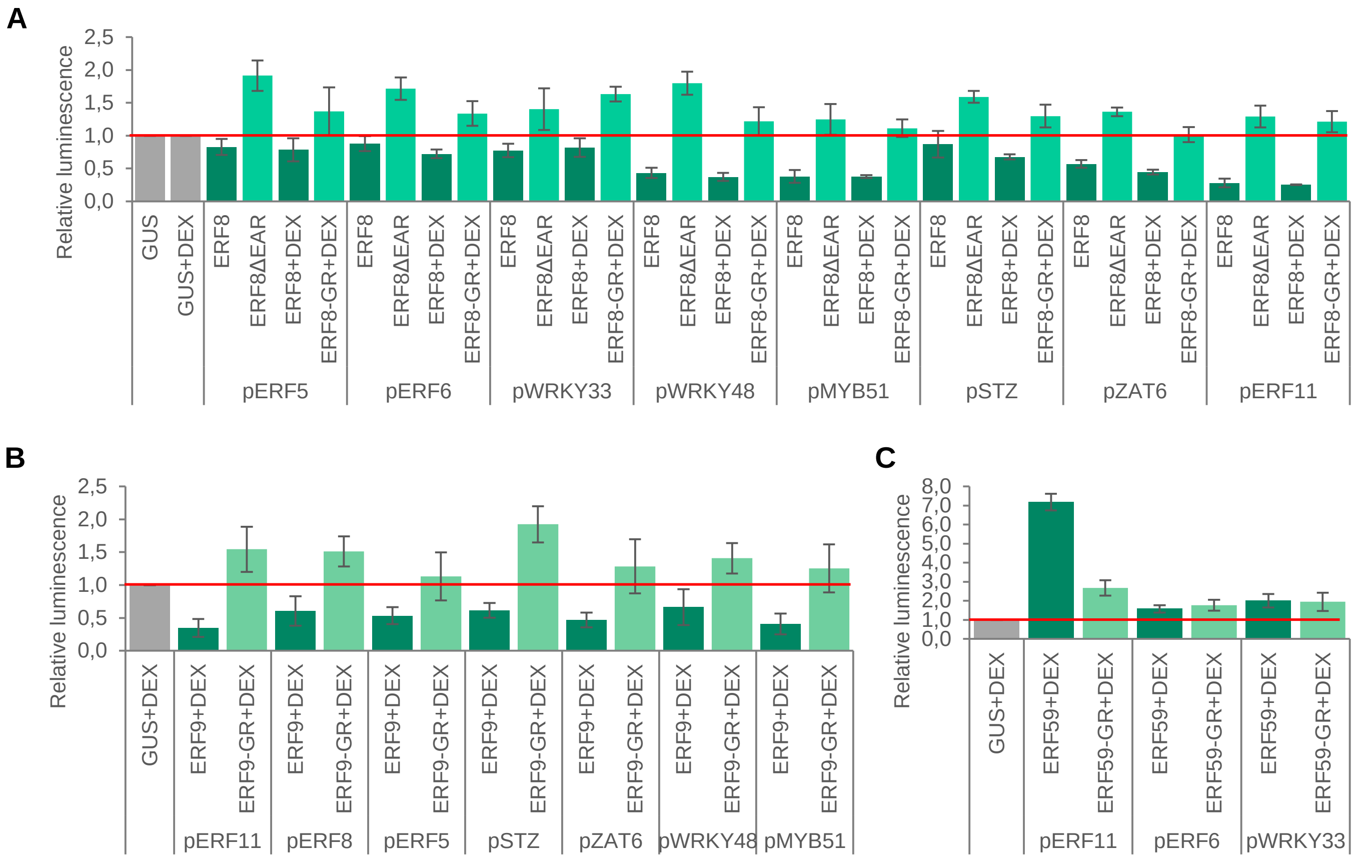

Appendix Figure S24 – Transient expression assays to assess the effect of adding a GR-domain.

A, B, C The effect of ERF8 (dark green) and ERF8ΔEAR (light green) (A), of ERF9 (dark green) and ERF9-GR (light green) (B) and of ERF59 (dark green) and ERF59-GR (light green) (C) on target genes identified in this study, after addition of 10 µM DEX for 4 h. The relative luminescence was calculated relative to the control, 35S::GUS (gray). n = 4 biological repeats.

Data information: Data are represented as mean ± SEM, n = 3 independent experiments.

Appendix Table S3 – Confirmed regulatory interactions between 20 genes encoding transcription factors.

A large-scale expression analysis was performed in which putative direct targets were identified. The measured expression values, the FDR corrected p-values, the first time point at which the gene is significantly differentially expressed, the average luminescence in the transient luciferase assays (TEAs) and the results from a large-scale promoter occupancy dataset (DAP-seq; O'Malley et al., 2016) are given. FC = Fold change.

Promotor	Regulator	$Log_2(FC)$	FDR	Time point	TEA	DAP-seq
ERF1	ERF59	0.90	0.019103	1h	1.49	NA
ERF1	ERF8	0.61	0.016902	1h	0.79	✓
ERF1	ERF98	0.62	0.071438	1h	2.37	NA
ERF1	WRKY48	0.94	0.020846	1h	1.36	NA
ERF11	ERF59	2.68	0.070405	1h	15.38	NA
ERF11	ERF8	2.12	0.028033	1h	0.58	✓
ERF11	ERF9	2.40	0.041776	1h	0.90	NA
ERF5	ERF8	0.79	0.020526	1h	0.55	✓
ERF5	ERF9	0.86	0.044109	1h	0.85	NA
ERF5	ERF98	1.36	0.040165	1h	1.43	NA
ERF59	ERF6	2.03	0.01408	1h	1.47	NA
ERF59	ERF98	2.24	0.050572	1h	1.35	NA
ERF6	ERF59	2.43	0.019147	1h	1.52	NA
ERF6	ERF8	1.90	0.030811	1h	0.70	✓
ERF8	ERF9	-0.57	0.077533	2h	0.46	NA
MYB51	ERF9	0.79	0.098944	1h	0.56	NA
MYB51	ERF6	1.20	0.001165	4h	1.36	NA
RAP26L	ERF98	2.98	0.003078	1h	1.42	NA
RAP26L	WRKY15	2.60	0.094323	2h	1.22	✓
RAP26L	ERF8	2.91	0.030273	2h	0.74	✓
STZ	ERF59	3.22	0.006443	1h	1.68	NA
STZ	ERF8	3.31	0.000317	1h	0.48	×
STZ	ERF9	2.77	0.005192	1h	0.43	NA
STZ	ERF6	1.91	0.09159	4h	1.15	NA
STZ	ZAT6	1.87	0.065485	1h	0.49	NA
STZ	ERF2	3.53	0.025831	4h	1.38	×
STZ	ERF11	2.12	0.015696	2h	0.82	×
STZ	WRKY15	2.75	0.008891	2h	1.32	✓
WRKY15	ERF8	0.97	0.003826	1h	0.73	✓
WRKY30	ERF98	3.23	0.078848	1h	1.52	NA
WRKY30	ERF59	-3.62	0.079938	4h	1.65	NA
WRKY33	ERF8	1.03	0.040496	1h	0.71	×
WRKY33	ERF59	1.27	0.045276	1h	1.87	NA
WRKY40	ERF8	1.63	0.027366	1h	0.64	×
WRKY40	ERF9	1.61	0.045276	1h	0.77	NA
WRKY40	ERF98	2.28	0.016902	1h	1.24	NA
WRKY48	ERF9	2.33	0.011796	1h	0.59	NA
WRKY48	ERF98	3.38	0.001349	1h	1.39	NA
WRKY48	ERF8	2.54	0.006443	1h	0.40	×
WRKY6	ERF8	0.87	0.01733	1h	0.60	✓
WRKY6	ERF9	0.72	0.046289	1h	0.78	NA
WRKY6	ERF98	1.21	0.0013	1h	1.20	NA
ZAT6	ERF8	1.80	0.033792	1h	0.50	×
ZAT6	ERF9	1.56	0.08971	1h	0.77	NA
ZAT6	ERF59	2.19	0.079281	2h	2.20	NA

Appendix Table S4 – The additional confirmed regulatory interactions between 20 genes encoding transcription factors as a result of the combination of two transcription factors.

A large-scale expression analysis was performed in which putative direct targets were identified of which 36 were confirmed with TEAs. An additional 23 regulatory interactions were confirmed when evaluating the transactivation capacities of two transcription factors on one target gene with TEAs. The listed regulators can thus regulate the promoter gene in combination with another necessary transcription factor.

Regulator	Promotor
ERF1	WRKY15
ERF1	WRKY6
ERF2	ERF9
ERF59	WRKY15
ERF59	WRKY48
ERF6	WRKY15
ERF6	WRKY48
ERF6	ZAT6
ERF9	ERF6
ERF9	WRKY15
ERF98	ERF6
ERF98	MYB51
ERF98	STZ
ERF98	WRKY15
ERF98	WRKY33
ERF98	ZAT6
STZ	ERF9
STZ	WRKY15
WRKY15	ERF1
WRKY15	WRKY6
WRKY15	ZAT6
WRKY48	ERF6
WRKY48	WRKY33

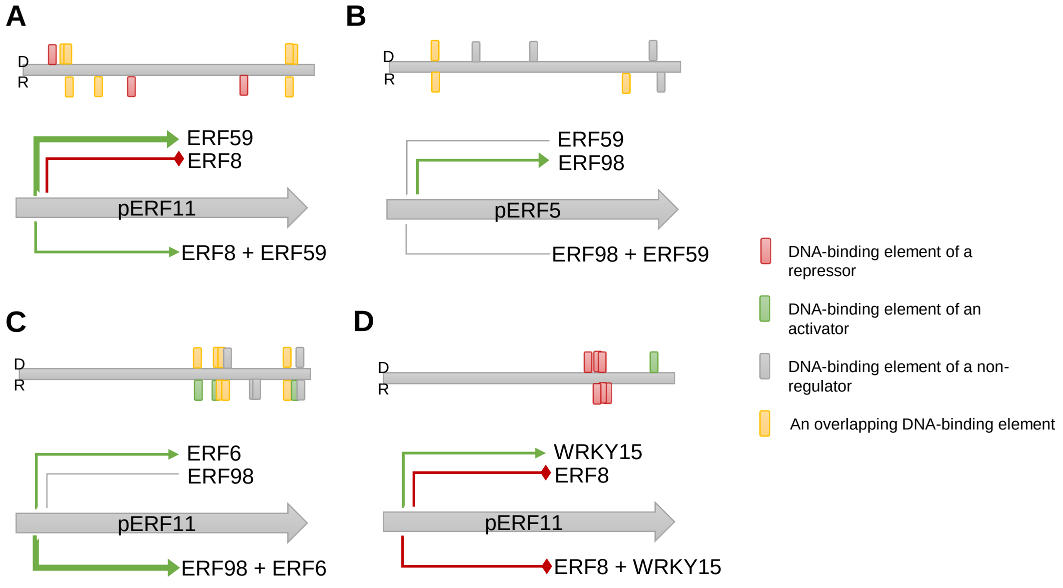

Appendix Figure S25 – Five different effects of two TFs on a target gene and the estimated position of the DNA-binding elements on the target promoter.

A, B, C, D The upper panel are schematic representations of the target promoter with its DNA-binding elements is given on either the direct (D) or reverse strand (R). The lower panel each time represents the corresponding TEA's, as described in Fig 7. Green arrows represents activation, red repression and gray absence of regulation.
