# Supplementary material for: From network to phenotype: the dynamic wiring of an Arabidopsis transcriptional network induced by osmotic stress
Source: Mol Syst Biol. 2017 Dec 21;13(12):961. doi: 10.15252/msb.20177840 (PMC5740496; doi:10.15252/msb.20177840)
Supplement: Supplementary file 2 — Expanded View Figures PDF [file MSB-13-961-s002.pdf]

Expanded View Figures

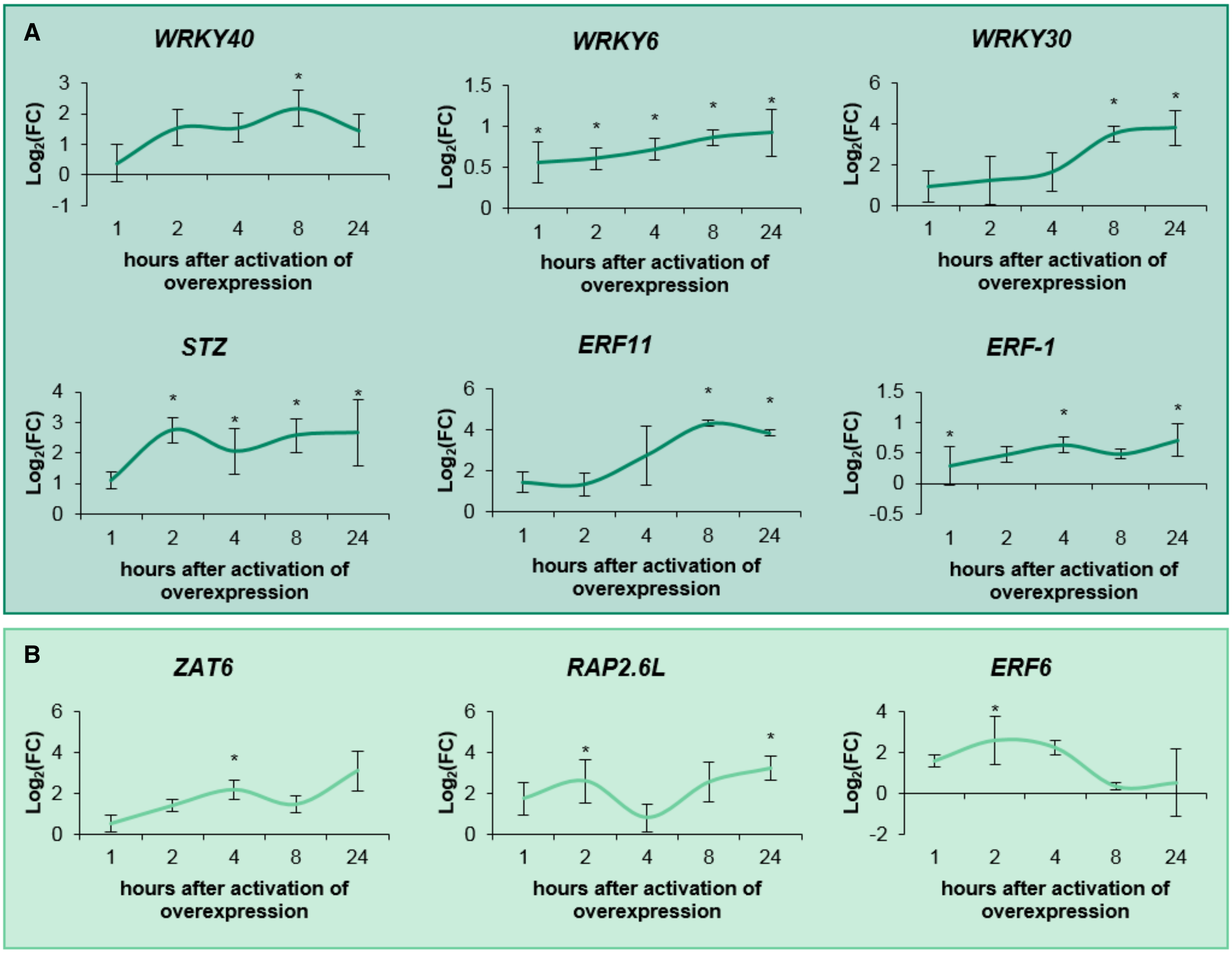

Figure EV1. Expression profiles of WRKY15 target genes.

Expanding leaf tissue (third leaf – 15 DAS) of WRKY15-GR was harvested 1 h, 2 h, 4 h, 8 h, and 24 h after transfer to dexamethasone. Expression values were normalized against the control line.

A WRKY15 target genes (significantly differentially expressed during one time point) that showed a gradually increasing expression pattern.

B WRKY15 target genes that showed an oscillating expression pattern.

Data information: data are presented as mean ± SEM, $n = 3$ independent experiments, *FDR < 0.1 (mixed model analysis, user-defined Wald tests).

 © 2017 The Authors

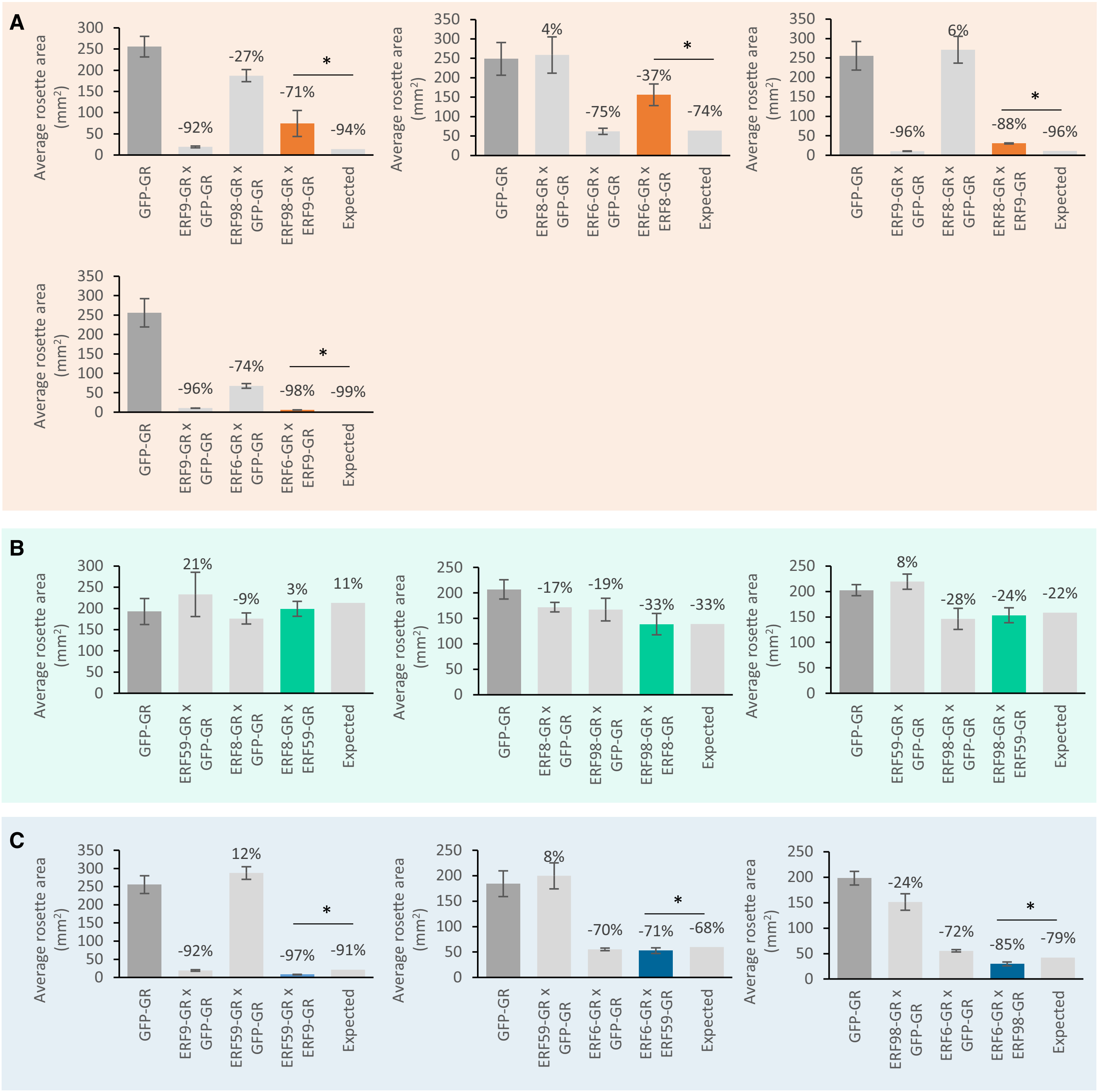

Figure EV2. Phenotypic analysis of crosses between two GOF lines of the core network.

A–C All members of the core network were crossed with either each other, referred to as double cross, or with the control line (GFP-GR), referred to as single cross and were germinated and grown on dexamethasone (n = 8 plants). At 22 DAS, the projected rosette area of the double and single cross and the GFP-GR was measured. Double crosses resulted in a synergistic (A), an additive (B), or a negative (C) phenotype and are depicted in orange, green, and blue connection, respectively.

Data information: Data are presented as mean ± SEM, n = 3 independent experiments, *$P < 0.05$.
