# Supplementary material for: From network to phenotype: the dynamic wiring of an Arabidopsis transcriptional network induced by osmotic stress
Source: Mol Syst Biol. 2017 Dec 21;13(12):961. doi: 10.15252/msb.20177840 (PMC5740496; doi:10.15252/msb.20177840)
Supplement: Supplementary file 3 — Table EV1 [file MSB-13-961-s003.pdf]

Table EV1 - The induction of 20 genes encoding TFs upon mannitol-induced stress in expanding leaf tissue.

The expression level of 20 genes encoding TFs was measured in wild-type plants at 15 DAS, 20 min, 40 min, 1 h, 2 h, 4 h, 8 h, 12 h, 16 h, 24 h and 48 h upon mannitol treatment in both control and mannitol conditions. Data information: Data are presented as mean ± SEM, n = 4 independent experiments. FC = Fold change. FDR-corrected p-values, Student's t-test.

	20'		40'		1h		2h		4h		8h		12h		16h		24h		48h	
	Log(FC)	FDR	Log(FC)	FDR	Log(FC)	FDR	Log(FC)	FDR	Log(FC)	FDR	Log(FC)	FDR	Log(FC)	FDR	Log(FC)	FDR	Log(FC)	FDR	Log(FC)	FDR
ERF-1	0.13	0.8189	0.57	0.6942	1.62	0.0232	2.05	0.0182	1.14	0.0237	0.75	0.0242	0.71	0.0437	0.6	0.3341	0.62	0.0437	0.24	0.7208
ERF2	-0.23	0.8200	0.57	0.7636	1.64	0.0536	2.65	0.0212	2.34	0.0296	2.48	0.0455	2.12	0.0089	1.05	0.0373	1.28	0.0965	0.68	0.3948
ERF5	0.62	0.6049	1.44	0.5054	3.9	0.0051	4.44	0.0107	3.99	0.0024	2.92	0.0009	2.88	0.0011	2.89	0.0032	3.52	0.0187	2.18	0.0094
ERF6	0.29	0.9211	1.47	0.6395	3.8	0.0101	5.25	0.0162	4.04	0.0279	2.69	0.1021	0.96	0.1174	1.98	0.0498	2.47	0.0372	2.31	0.0867
ERF8	0.15	0.7040	0.27	0.5759	0.39	0.1574	1.13	0.0028	0.94	0.0258	0.62	0.0115	0.5	0.0213	0.33	0.0743	0.28	0.4116	0.35	0.0638
ERF9	-0.02	0.9803	-0.44	0.2742	0.04	0.9725	1.12	0.0801	0.64	0.0844	0.73	0.0743	1	0.0498	0.53	0.3601	0.86	0.0784	0.27	0.9067
ERF11	0.37	0.8189	1.04	0.8189	3.77	0.0705	5.73	0.0202	6.22	0.0026	5.23	0.0100	3.66	0.0022	2.94	0.0182	4.32	0.0085	5.34	0.0015
ERF59	-1.05	0.2710	-0.37	0.7892	-0.92	0.6968	2.77	0.1487	3.17	0.0999	4.52	0.0715	4.87	0.0191	1.96	0.0437	2.25	0.2719	1.11	0.4966
ERF98	2.19	0.1817	1.41	0.6017	4.87	0.0117	7.32	0.0007	6.32	0.0008	5.25	4.9E-5	5.58	0.0006	4.86	0.0004	3.97	0.0321	3.1	0.0245
RAP2.6L	0.26	0.9067	0.06	0.9834	0.12	0.9640	0.78	0.7635	5.32	0.0029	4.1	0.0167	4.24	0.0142	2.55	0.0793	3.71	0.0237	2.43	0.3890
WRKY6	-0.05	0.9067	0.07	0.6905	0.61	0.0236	1.81	0.0048	2.22	0.0082	1.96	0.0283	1.64	0.0117	1.29	0.0271	1.48	0.0022	1.1	0.0437
WRKY15	0.05	0.8189	0.12	0.7635	0.7	0.0212	1.88	0.0324	1.71	0.0103	1.06	0.0518	1.1	0.0057	0.92	0.0165	1.03	0.0611	0.75	0.0616
WRKY28	0.93	0.8635	-0.11	0.7262	0.07	0.0182	1.33	0.0024	2.56	0.0042	3.47	0.0022	3.65	0.0004	2.7	0.0035	2.44	0.0057	1.05	0.0165
WRKY30	0.34	0.9510	0.23	0.9364	2.07	0.3631	5.58	0.0163	6.41	0.0157	8.41	0.0018	5.55	0.0022	7.12	0.0237	4.78	0.0048	4.2	0.0261
WRKY33	0.27	0.9510	0.86	0.9067	2.59	0.2297	3.91	0.0094	3.3	0.0147	2.46	0.0456	2.12	0.0031	1.95	0.0260	2.33	0.0022	1.69	0.0085
WRKY40	0.27	0.7635	1.64	0.9767	3.03	0.9803	5.59	0.0904	5.19	0.0103	4.51	0.0212	2.38	0.0167	2.39	0.0995	2.86	0.0042	1.71	0.4712
WRKY48	0.34	0.8635	-0.5	0.9364	0.32	0.0743	2.99	0.0330	3.47	0.0023	3.89	0.0015	1.91	0.0004	1.08	0.0014	2.79	0.0057	2.44	0.0069
MYB51	0.2	0.8411	0.62	0.6905	2.36	0.0212	3.83	0.0048	4.22	0.0048	3.69	0.0066	2.8	0.0022	1.62	0.0048	2.16	0.0051	1.52	0.0081
STZ	0.21	0.8721	1.23	0.5501	4.01	0.0311	5.84	0.0227	5.01	0.0296	3.49	0.0264	2.12	0.0022	1.76	0.0182	2.93	0.0216	1.78	0.0118
ZAT6	0.54	0.8635	1.38	0.8244	4.41	0.8635	5.11	0.1725	4.16	0.0801	4.02	0.0580	2.33	0.0692	2.07	0.3757	3.39	0.0082	2.29	0.0182
