# Supplementary material for: From network to phenotype: the dynamic wiring of an Arabidopsis transcriptional network induced by osmotic stress
Source: Mol Syst Biol. 2017 Dec 21;13(12):961. doi: 10.15252/msb.20177840 (PMC5740496; doi:10.15252/msb.20177840)
Supplement: Supplementary file 4 — Table EV2 [file MSB-13-961-s004.pdf]

Table EV2 – Overview of the phenotypic analysis of the 20 TFs under normal conditions.

The outcome of the phenotypic analysis of the two or three independent GOF lines and the LOF line is given. A plus (+) and minus (-) represents a significant larger and smaller rosette area at 22DAS compared to the control, respectively. If no significant changes of the rosette area were observed a “0” is indicated and when a line was not present “NA” (Not Applicable) is indicated. The final outcome of the growth function of the TF is given considering that at least two independent GOF lines or the LOF line showed a growth phenotype and that the GOF and LOF lines did not show a contrasting growth-regulating function.

Gene	Phenotype GOF line			Phenotype LOF line	Growth function
	#1	#2	#3		
ERF-1	-	+	0	0	Inconclusive
ERF2	+	0	NA	-	Activator
ERF5	0	-	NA	0	Inconclusive
ERF6	-	-	NA		Repressor
ERF8	0	0	0	+	Repressor
ERF9	-	-	-	0	Repressor
ERF11	-	-	NA	+	Repressor
ERF59	+	+	0	0	Activator
ERF98	-	-	-	0	Repressor
RAP2.6L	0	0	NA	-	Activator
STZ	0	0	NA	-	Activator
ZAT6	0	0	NA	0	None
MYB51	0	0	NA	0	None
WRKY6	NA	NA	NA	-	Activator
WRKY15	-	-	-	-	Inconclusive
WRKY28	-	0	NA	0	Inconclusive
WRKY30	NA	NA	NA	+	Inconclusive
WRKY33	-	+	0	0	Inconclusive
WRKY40	NA	NA	NA	0	Inconclusive
WRKY48	0	NA	NA	0	Inconclusive
