# Supplementary material for: From network to phenotype: the dynamic wiring of an Arabidopsis transcriptional network induced by osmotic stress
Source: Mol Syst Biol. 2017 Dec 21;13(12):961. doi: 10.15252/msb.20177840 (PMC5740496; doi:10.15252/msb.20177840)
Supplement: Supplementary file 5 — Table EV3 [file MSB-13-961-s005.pdf]

Table EV3 – Expression values of 20 genes encoding TFs in published transcriptomic datasets.

Whole shoots or specific leaves were harvested upon different abiotic or biotic stress treatments according to the specifics as described in Windram et al., 2012, Dubois et al., 2017, McGrath et al., 2005 and Matsui et al., 2008. “null”: not present on the microarray, “UP”: upregulated in the Windram et al., 2012 dataset over the 24 time points, “Not DE”: not differentially expressed in the Windram et al., 2012 dataset over the 24 time points. The significant values according to the calculations of the corresponding manuscript are highlighted in green.

	Windram et al., 2012		Dubois et al., 2017				McGrath et al, 2005		Matsui et al., 2008							
	B. Cinerea	Senescence	3 days upon mild drought				MeJA	A. Brassicicola	NaCl		Dehydratation stress		Cold		ABA	
			4h	12h	20h	4h'	6h	6h	2h	10h	2h	10h	2h	10h	2h	10h
ERF-1	UP	UP	-0.37	-0.01	0.15	-0.35	3.69	2.28	2.41	2.73	0.55	0.46	0.85	-0.54	1.93	2.32
ERF2	Not DE	UP	-0.16	0.01	0.98	-0.75	3.53	2.17	1.83	1.07	0.67	-0.54	1.20	-0.89	0.76	-1.17
ERF5	UP	Not DE	0.59	-0.34	0.60	-1.65	4.92	0.63	0.73	0.16	1.27	0.58	-0.41	-1.14	1.23	-0.06
ERF6	UP	Not DE	0.10	0.04	-0.35	-0.79	1.58	3.39	2.09	1.34	2.21	0.74	2.37	0.67	1.44	-0.40
ERF11	UP	UP	-0.45	0.04	1.04	-4.24	null	null	1.55	1.18	2.79	1.77	1.67	0.10	2.08	-0.14
ERF8	UP	UP	0.22	0.25	1.12	0.34	0.97	1.08	2.32	2.54	2.52	2.85	-0.06	-0.40	0.53	0.43
ERF9	Not DE	Not DE	0.25	0.04	0.74	0.16	2.69	2.62	1.66	0.92	2.06	2.03	0.12	0.01	0.30	-0.58
ERF59	UP	Not DE	-1.08	-2.71	-5.80	-0.78	5.66	2.70	null	null	null	null	null	null	null	null
ERF98	UP	Not DE	null	null	null	null	2.82	14.18	null	null	null	null	null	null	null	null
RAP2.6L	UP	UP	-2.31	-0.82	-0.86	-0.62	null	null	1.93	3.07	0.51	2.82	0.59	0.81	2.25	3.60
MYB51	UP	Not DE	0.38	-0.50	0.76	-0.69	3.69	6.43	0.89	0.58	1.14	-0.10	1.47	-0.11	0.12	-0.17
STZ	UP	UP	-0.79	-0.68	0.53	-1.49	3.41	3.58	1.97	1.77	2.33	2.67	1.95	1.54	2.15	1.05
ZAT6	UP	UP	-0.91	-1.31	-0.57	-1.44	540.11	0.95	2.76	3.21	2.34	3.78	1.30	1.68	2.31	3.07
WRKY28	UP	UP	-1.40	0.22	1.90	-1.25	2.24	3.85	3.64	3.78	2.83	4.20	2.41	1.60	1.46	1.29
WRKY33	UP	UP	-0.04	-0.37	0.31	-0.90	null	null	2.67	2.58	2.54	2.21	2.14	0.60	1.33	0.84
WRKY15	UP	UP	-0.06	-0.25	0.29	-0.37	1.42	1.52	2.04	2.29	1.54	1.89	0.88	0.07	1.28	2.04
WRKY48	UP	UP	-1.48	-1.46	-1.28	-2.44	1.11	1.74	1.57	1.46	2.15	1.55	1.21	1.06	2.48	1.84
WRKY6	UP	UP	0.15	0.18	0.33	-0.27	1.63	1.51	1.81	2.03	1.21	1.64	1.28	1.41	1.71	2.56
WRKY30	UP	UP	-2.28	-4.17	-1.36	-3.35	3.06	50.80	4.20	4.22	4.11	3.99	1.26	0.38	-0.09	-0.06
WRKY40	UP	UP	-1.42	-0.86	0.08	-1.66	2.56	7.51	3.74	2.98	3.48	2.48	1.69	0.24	2.84	0.94
